# Transcription factor Sp1 transcriptionally enhances GSDME expression for pyroptosis

**DOI:** 10.1038/s41419-024-06455-6

**Published:** 2024-01-18

**Authors:** Jiasong Pan, Yuanyuan Li, Wenqing Gao, Qizhou Jiang, Lu Geng, Jin Ding, Suhua Li, Jixi Li

**Affiliations:** 1 Department of Neurology, Huashan Hospital, State Key Laboratory of Genetic Engineering and School of Life Sciences, Fudan University, Shanghai, China; 2 Division of Natural Science, Duke Kunshan University, Jiangsu, China; 3 Clinical Cancer Institute, Center for Translational Medicine, Naval Medical University, Shanghai, China

**Keywords:** Cell death, Transcriptional regulatory elements

## Abstract

Gasdermin-E (GSDME), the executioner of pyroptosis when cleaved by caspase 3, plays a crucial role in tumor defense and the response to chemotherapy drugs in cells. So far, there are poorly known mechanisms for the expression regulation of GSDME during cell death. Here, we identify the transcription factor Sp1 (Specificity protein 1) as a positive regulator of GSDME-mediated pyroptosis. Sp1 directly interacts with the GSDME promoter at −36 ~ −28 site and promotes GSDME gene transcription. Further, Sp1 knockdown or inhibition suppresses GSDME expression, thus reducing chemotherapy drugs (topotecan, etoposide, doxorubicin, sorafinib and cisplatin) induced cell pyroptosis. The regulation process synergizes with STAT3 (Signal transducer and activator of transcription 3) activity and antagonizes with DNA methylation but barely affects GSDMD-mediated pyroptosis or TNF-induced necroptosis. Our current finding reveals a new regulating mechanism of GSDME expression, which may be a viable target for the intervention of GSDME-dependent inflammatory diseases and cancer therapy.

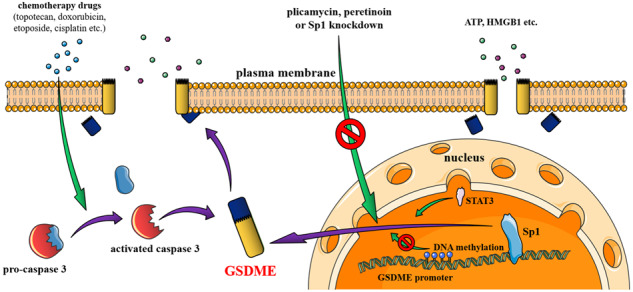

## Introduction

Cells can undergo pyroptosis in response to pathogen infection, cellular stress, and immune cell-mediated attack, essential for immune defense against microbial infections and tumorigenesis [1, 2]. Previous studies have revealed that primary members of the human gasdermin family, comprising GSDMA, GSDMB, GSDMC, GSDMD, and GSDME, are the pyroptotic executors and share a similar activation mechanism. Activated inflammatory caspases 1, 3, 4, 5, 8, or 11 cleave gasdermins, thus releasing a cytotoxic N-terminal domain from the self-inhibitory structure during pyroptosis [3–6]. This domain binds to membrane lipids (cardiolipin and phosphatidylserine), oligomerizes, and forms pores, resulting in cell membrane rupture and cytokines release [7–9].

GSDME-dependent pyroptosis has drawn increasing attention in recent years. In tumor immunity, when cancer cells endogenously or extracellularly express GSDME, activated caspase 3 induced by various chemotherapy drugs can further cleave and activate GSDME, triggering ‘secondary apoptosis’ [10]. Tumor cells also undergo caspase 3-GSDME axis-mediated pyroptosis while attacked by NK cells, effector T cells, or CAR-T cells [11, 12]. Inflammatory factors released from the disrupted cell membrane, like ATP and HMGB1, can induce inflammatory responses and enhance the efficiency of tumor clearance [11–13]. However, excessive pyroptosis may carry the risk of side effects, such as cytokines release syndrome [12]. Caspase 3-GSDME axis-mediated pyroptosis also triggers or promotes the symptom of many inflammatory diseases. For example, cytokines released from GSDME-mediated pyroptotic intestinal epithelial cells (IEC) in Crohn’s disease aggravate colitis [14]; liver injury caused by bile acid accumulation is associated with GSDME-mediated pyroptosis [15]; GSDME is also involved in pneumonia symptoms caused by viruses such as H7N9 and COVID-19 [16].

In many cases, high expression of GSDME switches cell death mode from apoptosis to pyroptosis, causing a robuster inflammatory response [10]. Hence, multiple tumor cells could enhance immune escape by reducing the expression of GSDME [11]. Therefore, revealing the expression regulation mechanism of GSDME is of great significance. Previous reports showed that the transcription factor STAT3 upregulates GSDME expression in atherosclerosis [17]. Also, DNA methylation is shown to suppress GSDME transcription by blocking transcription factor binding [18]. However, the detailed mechanism for GSDME transcriptional regulation remains poorly understood.

Here, we identified that the transcription factor Sp1 positively regulates GSDME transcriptional regulation by binding the specific site in the GSDME promoter. Knocking down or drug inhibition of GSDME ameliorates chemotherapy drug-induced pyroptosis. The regulation process synergizes with STAT3 activity and antagonizes with DNA methylation but barely affects GSDMD-mediated pyroptosis or TNF-induced necroptosis. This provides a new sight for inflammatory disease or drug therapy via GSDME transcriptional regulation.

## Results

### Transcription factor Sp1 binds to the promoter region of GSDME and promotes downstream gene expression

**Fig. 1:**
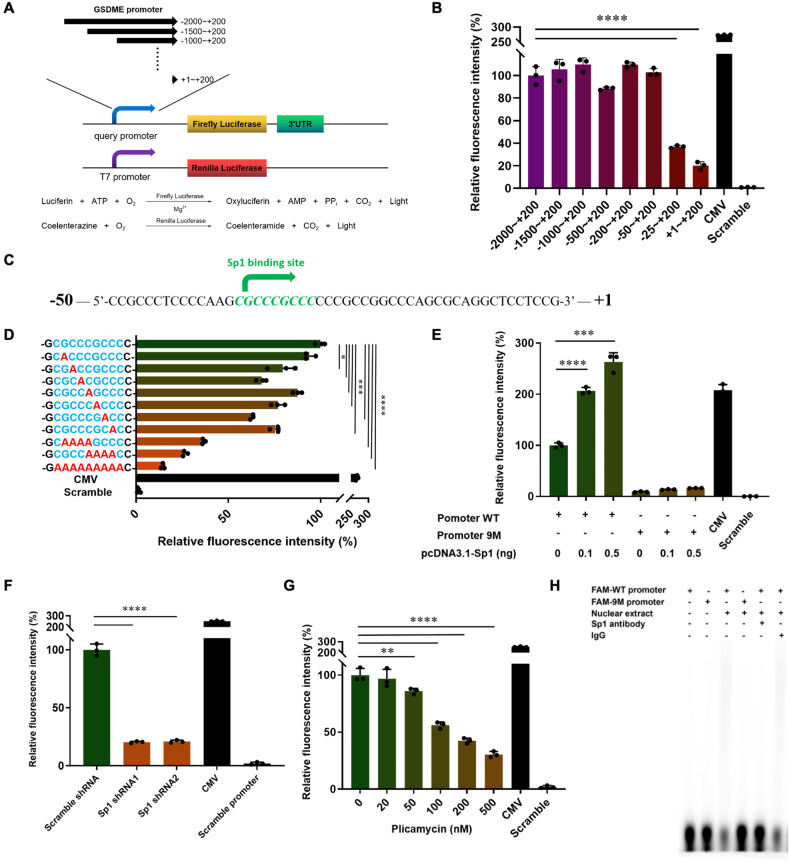
Transcription factor Sp1 binds to the promoter region of GSDME and promotes downstream gene expression. **A**, **B** Analysis for transcription activity of GSDME promoter in HeLa cells after transfection of GSDME promoter truncations-pGL-basic plasmids. The transcriptional activity was measured by firefly luciferase expression driven by the query promoters. **C** Schematic of the -50 ~ + 1 sequence of GSDME promoter. The Sp1-binding consensus sequence is shown in green. **D** Analysis of GSDME promoter activity in the luciferase reporter assay and schematics of the Sp1 motif mutations. HeLa cells were transfected with the pGL-basic containing WT (wild-type)/mutated GSDME promoter. Reporter gene activation was measured by the luciferase activity. **E** Analysis of GSDME promoter activity after HeLa cells were co-transfected with the WT/9M (nine mutated nucleotides) GSDME promoter-pGL-basic plasmid and indicated amounts of Sp1 plasmid. **F** Analysis of GSDME promoter activity after HeLa cells were co-transfected with the WT GSDME promoter-pGL-basic plasmid and the control vector or Sp1 shRNA. **G** Analysis of GSDME promoter activity after HeLa cells were transfected with the WT GSDME promoter-pGL-basic plasmid and treated by indicated amounts of plicamycin. In each luciferase reporter assay above, the luciferase activity is measured 48 h after transfection and is normalized to Renilla luciferase activity produced by pRL-TK plasmid. **H** The direct binding of Sp1 to the GSDME promoter was determined by EMSA assay, which was performed with nuclear extracts from HeLa cells using FAM-labeled 30 bp dsDNA containing wild-type or mutated Sp1 binding sites as probes. The indicated binding reaction mixture was separated on a 6% polyacrylamide gel and detected with a phosphor imager.

In order to identify the critical promoter region that regulates GSDME expression, luciferase constructs harboring different truncations of the candidate GSDME promoter are generated, in which the reporter luciferase activity is converted into the light signal by the specific substrate to represent the promoter activity (Fig. 1A). The promoter activity of +1 ~ + 200 truncation shows a significant decrease compared to −50 ~ + 200 in HeLa cells, indicating that a functional promoter in the −50 ~ + 1 region harbors a critical transcription binding site (Fig. 1B). Using ALGGEN-PROMO software, the zinc-finger transcription factor Specific protein 1 (Sp1) is predicted to be the putative transcription factor that binds the −36 ~ −28 site in the GSDME promoter (Fig. 1C). Sp1 belongs to the Sp/KLF family, which performs high affinities to GC-rich promoter elements, including CACCC-boxes and GC-boxes [19, 20]. Sp1 regulates numerous gene expression related to cell proliferation and death [21–23]. All-nine-nucleotide and the four-nucleotide mutation within the putative Sp1 binding site strongly inhibit GSDME promoter activation (Fig. 1D). In contrast, single-nucleotide mutations partially inhibit the activity (Fig. 1D), confirming that the −36 ~ −28 site plays a critical role in GSDME expression. To verify that Sp1 is the crucial transcription factor of GSDME transcription, we overexpressed Sp1 in HeLa cells. Expression of luciferase driven by GSDME promoter containing wild-type Sp1 binding site obviously increases in response to Sp1 overexpression. However, promoter activation is completely abrogated upon all-nucleotide mutation of the Sp1 binding site (Fig. 1E). Further, knocking down Sp1 by shRNA decreases the luciferase expression level in cells (Fig. 1F). Also, plicamycin, a small molecular drug that inhibits Sp1 by inducing proteasome-dependent degradation [24], suppresses luciferase expression in a concentrate-dependent manner (Fig. 1G). Next, to verify the direct DNA binding of Sp1 to its binding site, an EMSA assay was conducted. Anti-Sp1 antibody or Sp1 binding site mutation prevents the super-shifted complex formation observed in the presence of IgG (Fig. 1H). Thus, Sp1 is the transcription factor for GSDME promoter activity by binding its −36 ~ −28 site.

### Sp1 regulates GSDME expression

**Fig. 2:**
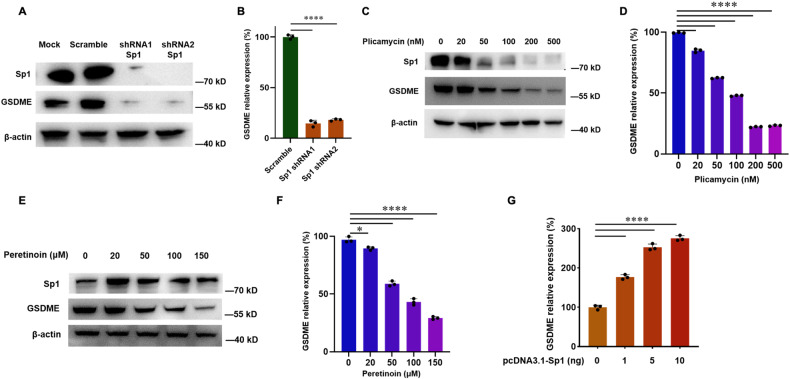
Sp1 regulates GSDME expression. **A**, **C**, and **E** HeLa cells transfected by the control or Sp1 shRNA or treated with the indicated amounts of inhibitors, plicamycin or peretinoin, were harvested and lysed in the RIPA lysis buffer. Western blotting analysis of lysates from the cells shows the Sp1, GSDME, and β-actin levels. **B**, **D**, and **F** HeLa cells transfected by the control or Sp1 shRNA, or treated with the indicated amounts of inhibitors plicamycin or peretinoin. qPCR analysis shows GSDME mRNA level, normalized by β-actin level. **G** qPCR analysis result of GSDME mRNA level in HeLa cells transfected by indicated amounts of Sp1 plasmids.

As Sp1 binds to the GSDME promoter and activates downstream reporter gene expression, we investigated if Sp1 regulates GSDME expression directly. Sp1 was knocked down by shRNA in HeLa cells, dramatically decreasing the mRNA and protein expression levels of GSDME (Fig. 2A, B). Plicamycin treatment also decreases GSDME expression in a concentrate-dependent manner in both HeLa and human liver cancer cell line Huh7 cells, consistent with the Sp1 knockdown results (Figs. 1G, 2C, D, S1A, B). Peretinoin, another Sp1 inhibitor that inhibits Sp1 activity by promoting Sp1 cross-linking and inactivation [25], shows the same effect (Figs. 2E, F, S1C, D). To exclude the direct effects of plicamycin or peretinoin on GSDME, we overexpressed HA-tagged GSDME in endogenous GSDME deficient HEK293T cells in an Sp1-independent manner. The result shows that ectopic expression of GSDME overcomes plicamycin or peretinoin induced GSDME depletion (Fig. S1E, F). Conversely, Sp1 overexpression increases GSDME mRNA level in HeLa cells (Fig. 2G). These data suggest that Sp1 acts as the positive transcriptional regulator of GSDME.

### Sp1 modulates GSDME-mediated pyroptosis via GSDME expression regulation

**Fig. 3:**
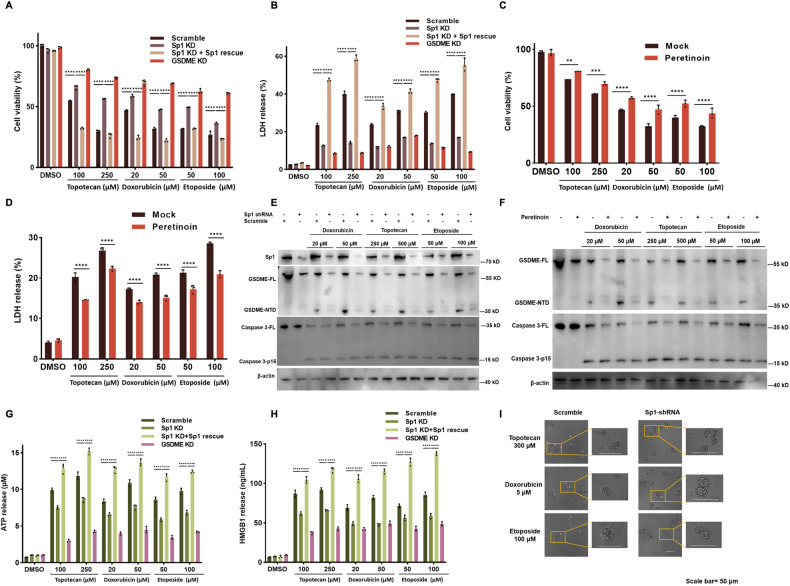
Sp1 modulates GSDME-mediated pyroptosis via GSDME expression regulation. **A**, **B** Scramble-shRNA, Sp1-shRNA, GSDME-shRNA, and Sp1-rescue expressed HeLa cells were treated with chemotherapy drugs topotecan, doxorubicin, or etoposide for 24 h, followed by cell death and LDH release analysis. **C**, **D** HeLa cells were treated with peretinoin for 24 h, followed by indicated chemotherapy drugs for another 24 h. Cell viability and LDH release were determined. **E**, **F** Western blotting analysis of the protein levels (Sp1, GSDME, cleaved GSDME, caspase 3, cleaved caspase 3, and β-actin) in WT, scramble-shRNA, Sp1-shRNA, or peretinoin-treated cell lines stimulated by indicated chemotherapy drugs for 24 h. **G**, **H** ATP and HMGB1 levels were measured in supernatant harvested from indicated cell lines treated by chemotherapy drugs. **I** The morphologies of chemotherapy drugs-induced death in scramble/Sp1-shRNA HeLa cells. Bubbing cells are shown with white arrows.

To better understand the biological function of Sp1 in GSDME-mediated pyroptosis, we assayed viability and LDH release of cells in pyroptotic stimulation after Sp1 knocking down. Topotecan, etoposide, and doxorubicin are chemotherapy drugs that interact with DNA and inhibit DNA topoisomerase [26–28]. They are known to kill cells via GSDME-mediated pyroptosis, while caspase 3 inhibitor Z-DEVD-FMK intervention rescues cell viability and GSDME cleavage (Fig. S2A, B) [10]. HeLa cell mortality and LDH release significantly decrease upon all three chemotherapy drugs stimulation after Sp1 knocking down. In Sp1-shRNA cells, shRNA-resistant Sp1 re-expression recovers cell death levels (Fig. 3A, B). Sp1 inhibitor peretinoin shows a similar effect that enhances tolerance to the drugs above (Fig. 3C, D). Inhibition of Sp1 by plicamycin also ameliorates cell death in Huh7 after treatment with the chemotherapy drugs sorafinib and cisplatin, respectively (Fig. S2C–F). In cells treated by Sp1-shRNA or Sp1 inhibitors, less cytotoxic GSDME N-terminal fragments are generated after stimulation because of GSDME transcriptional repression, resulting in drug resistance (Fig. 3E, F). Intriguingly, decreased protein level but not mRNA level of caspase 3 is also found in Sp1-shRNA or Sp1 inhibitor-treated cells. In contrast, the cleaved caspase 3 level is comparable (Figs. 3E, F, S3A, D), probably because Sp1 suppression slightly disturbs caspase 3 translation but barely affects caspase 3 activation. In GSDME-executed pyroptotic cancer cells, pores in the plasma membrane allow DAMP releases, such as ATP, HMGB1, and HSP70 [12]. We found much lower levels of ATP and HMGB1 in Sp1-shRNA pyroptotic supernatants after chemotherapy stimulation compared with the control group supernatants (Fig. 3G, H). Further, pyroptosis is characterized by cell swelling and membrane rupturing with many bubble-like protrusions. Evident characteristic large bubbles from the plasma membrane and cell swelling are found in dying control HeLa cells, whereas Sp1-shRNA dying cells present apoptotic performance, including cell shrinkage and packed apoptotic bodies (Fig. 3I). These observations suggest that Sp1 modulates GSDME-mediated pyroptosis by regulating the apoptosis-to-pyroptosis switch.

### Sp1-mediated GSDME expression can be affected by STAT3 and DNA methylation

**Fig. 4:**
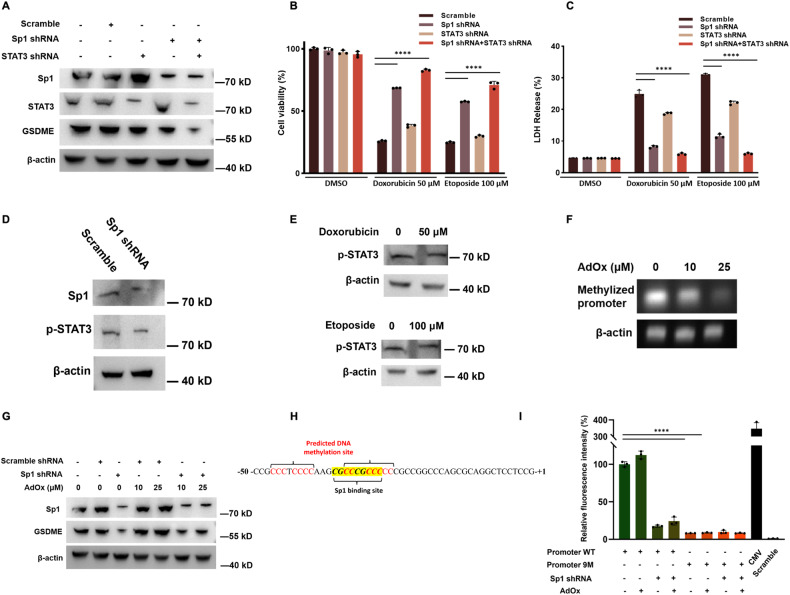
Sp1-mediated GSDME expression is affected by STAT3 and DNA methylation. **A** Western blotting analysis of the protein levels (Sp1, STAT3, GSDME, and β-actin) in scramble/Sp1/STAT3 shRNA in HeLa cells. **B**, **C** Scramble/Sp1/STAT3 shRNA or Sp1 plus STAT3 shRNA HeLa cells were treated with chemotherapy drugs doxorubicin or etoposide for 24 h, followed by cell death and LDH release analysis. **D**, **E** Western blotting analysis of the protein levels (Sp1, pSTAT3, and β-actin) in scramble/Sp1 shRNA or chemotherapy drug treatment in HeLa cells. **F** Genomic DNA was extracted and treated with sodium bisulfite. The methylation status of the region adjacent to the Sp1 binding site was evaluated by 19-cycle-PCR amplification and agarose gel electrophoresis. **G** Western blotting analysis of the protein levels (Sp1, GSDME, and β-actin) in scramble/Sp1-shRNA or AdOx-treated HeLa cells. **H** Schematics of the -50 ~ + 1 sequence of GSDME promoter. The Sp1-binding consensus sequence is shown in highlighted yellow, and the predicted methylation site is shown in red. **I** Analysis of GSDME promoter activity. HeLa cells were co-transfected with the WT/9M GSDME promoter-pGL-basic plasmid and scramble/Sp1 shRNA followed by AdOx treatment.

It has been demonstrated that transcription factor STAT3 upregulates GSDME expression [17]. The endogenous phosphorated STAT3 (pSTAT3) level elevates during ox-LDL or TNF treatment. pSTAT3 directly interacts with the GSDME promoter and initializes transcription [17]. Hence, we wondered about the relationship between Sp1 and STAT3 in GSDME regulation. The results show that either Sp1 or STAT3 knockdown by shRNA reduced GSDME expression (Fig. 4A). In contrast, double knockdown Sp1 and STAT3 further suppresses the GSDME level, compared to single knockdown (Fig. 4A). Moreover, cells transfected by Sp1-shRNA and STAT3-shRNA show higher tolerance to doxorubicin and etoposide than single knockdown (Fig. 4B, C). However, neither Sp1 knockdown nor chemotherapy drug treatment affects pSTAT3 expression level (Fig. 4D, E), indicating that Sp1 may maintain the constitutive GSDME expression, while pSTAT3 plays a role for additional expression in response to proliferation and inflammation signals. These results suggest a synergic function between Sp1 and STAT3.

DNA methylation has been found in the GSDME promoter region to block transcription factor binding and gene expression [18]. To reveal the connection between Sp1 and DNA methylation in GSDME regulation, we treated the control and Sp1-shRNA HeLa cells with AdOx, a DNA methylation inhibitor. AdOx reduces GSDME promoter methylation level, and the repression of GSDME expression in Sp1-shRNA is notably ameliorated after AdOx treatment (Fig. 4F, G). To further investigate the role of DNA methylation in GSDME expression regulation, we analyzed methylation sites using Methprimer software. Two CpG islands adjacent to the Sp1 binding region were predicted as potential methylation sites, indicating the DNA methylation of the GSDME promoter probably blocks Sp1 binding by a nearby steric hindrance, explaining how DNA methylation inhibitor overcomes Sp1 depletion (Fig. 4H). Moreover, AdOx fails to relieve the abrogative activity for the mutated GSDME promoter, suggesting that no more active transcription factors other than Sp1 exist under such conditions (Fig. 4I). These data indicate the antagonism between Sp1 and DNA methylation.

### Sp1 is dispensable for other necrosis-associated gene expressions

**Fig. 5:**
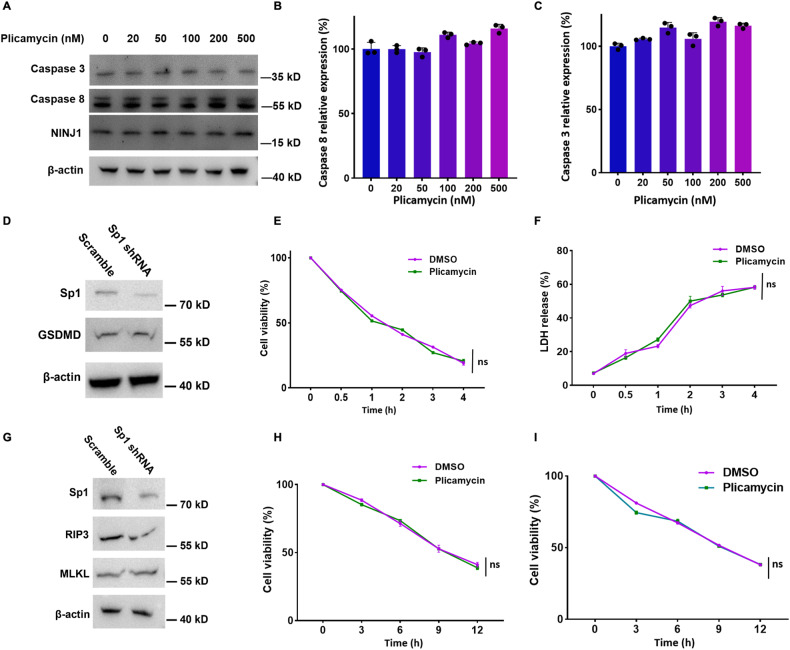
Sp1 is dispensable for other necrosis-associated gene expressions. **A** Western blotting analysis of lysates from Huh7 cells treated with indicated amount of plicamycin shows the protein expression levels of caspase 3, caspase 8, NINJ1, and β-actin. **B**, **C** qPCR analysis of caspase 3 and caspase 8 mRNA levels from Huh7 cells treated with the indicated amount of plicamycin, normalized by β-actin. **D** Western blotting analysis of lysates from iBMDM cells transfected with scramble/Sp1 shRNA. The protein expression levels of GSDMD and β-actin were detected. **E**, **F** iBMDM cells were treated with peretinoin for 24 h, followed by LPS and nigericin (Nig) stimulation for the indicated times. Cell viability and LDH release were determined, respectively. **G** Western blotting analysis of lysates from HT-29 cells transfected by scramble/Sp1 shRNA shows the protein expression levels of human RIP3, MLKL, and β-actin. **H**, **I** HT-29 and MEF cells were treated with peretinoin for 24 h, followed by TNF, Smac-mimetic, and Z-VAD-FMK stimulation at the indicated times. Cell viability was determined.

GSDME is the key to the apoptosis-to-pyroptosis switch, in which the original elements in the apoptosis pathway, including caspase 3, caspase 8, and NINJ1, also play a vital role in GSDME-mediated pyroptosis [10, 29]. To investigate whether Sp1 is necessary for the genes expression, Huh7 and SY5Y cells were treated with plicamycin. Despite the slight decrease of caspase 3 translation (Fig. 3F), we found no significant changes in caspase 3 or caspase 8 transcription, or in the protein expression of caspase 8 and NINJ1 (Figs. 5A–C, S3A–F). In innate immune cells such as macrophages, GSDMD-mediated pyroptosis is the primary manner of death in the stimulation of DAMPs or PAMPs [4, 5, 30, 31]. However, Sp1 inhibited by shRNA or plicamycin fails to alter GSDMD expression in immortal BMDM (iBMDM) cells (Figs. 5D and S3G). Moreover, the Sp1 inhibition does not affect cell viability or LDH release in iBMDM cells treated by the pyroptosis stimulators LPS and nigerin (Fig. 5E, F). Thus, Sp1 is dispensable for other pyroptosis-associated gene transcriptional expression.

Besides pyroptosis, necroptosis is another inflammatory cell death featured by membrane rupture and cytokine release when apoptosis is inhibited. Necroptosis is executed by RIP1/RIP3 necrosome formation and MLKL phosphorylation [32–34]. The expression levels of the executioner elements RIP3 and MLKL are unaffected by Sp1 shRNA or plicamycin treatment in HT-29 cells (Figs. 5G and S3H). Further, HT-29 and MEF cells processed TNF/Z-VAD/Smac-mimetic (TSZ) stimuli triggered necroptosis shows almost unchanged viability or LDH release after plicamycin treatment (Fig. 5H, I), suggesting Sp1 is dispensable for necroptosis process.

## Discussion

Pyroptosis is a double-edged sword that plays significant roles in anti-microbial and anti-cancer immune responses. As a pathogen and cancer defense, pyroptosis can be triggered by bacterial nucleic acid and flagellin in the canonical pathway or by LPS in the non-canonical pathway. The executioner protein GSDMD is cleaved and activated by caspase 1 or caspase 4/5/11, respectively [3–6]. GSDMD subsequently oligomerizes and forms pores on the membrane, inducing cell lysis and cytokine release. This process effectively activates innate immunity responses such as tumor cell death, macrophage recruiting, and IFNγ production [7–9]. Anti-cancer immune responses are primarily executed by GSDME-mediated pyroptosis. GSDME is the second detailed-studied member in the gasdermin family, performing similar oligomerization and pore-forming functions to GSDMD. The difference is that GSDME is cleaved by activated caspase 3 in chemotherapy drugs or immune cell attacking [10–12].

Our current study identified Sp1 as a transcriptional regulator for GSDME. Sp1 directly binds the −36 ~ −28 site of the GSDME promoter to initialize GSDME transcription. Sp1 increases GSDME expression to ensure pyroptosis occurs during cancer cells stimulated by chemotherapy drugs, consistent with the previous report that the expression of GSDME is essential to the apoptosis-to-pyroptosis switch (Figs. 1–5). Moreover, Sp1 knockdown or inhibition decreases GSDME transcriptional expression and further protects cells from chemotherapy drug attacks, with less cellular content release. The regulation process has a synergy with STAT3 and an antagonism with DNA methylation (Fig. 4).

Abnormal GSDME-mediated pyroptosis usually causes inflammatory diseases in cancer therapies [10–12]. Multiple chemotherapy drugs, including cisplatin, mitoxantrone, and doxorubicin, cause typical tissue damage via GSDME activation [10]. Overexpression of GSDME is one of the primary reasons because it causes pyroptosis out of regulation and releases excessive cytokines. Severe cytokine release syndrome can be triggered by CAR-T therapy as a result of excess pyroptotic cancer cells [12]. Therefore, proper regulation of the GSDME level is of vital importance. The intervention of the transcription process induced by Sp1 might be a potential therapeutic target for ameliorating the vice-effects of chemotherapy drugs and CAR-T therapy.

Further research is still required to gain insight into GSDME transcriptional regulation thoroughly. For example, the detailed crosstalk among GSDME transcription factors remains unclear; the method of avoiding typical tissue damage of chemotherapy drugs without reducing the effect remains to be studied. An explanation of these questions will offer a better understanding of anti-cancer immunity mechanisms and gasdermin physiological roles. We can also find more targets and therapies for treating cancer and inflammatory diseases.

## Methods and Materials

### Plasmid construction and RNA interference

Sp1 cDNA was kindly provided by Dr. Sudan He (Soochow University, China) and cloned into the pcDNA3.1( + )-HA vector. Human GSDME promoter and its truncations were amplified of cDNA from HeLa cells by PCR and then subcloned into the pGL3-basic vector. The Sp1-shRNA-resistant expression constructs mutated six nucleotides within the Sp1 shRNA targeting region without affecting the amino acid sequence. shRNA for RNA interference 5'-GGATGGTTCTGGTCAAATACA-3' for Sp1; 5'-GGGTCTCGGAAATTTAACATT-3' for STAT3 and 5'-GCGGTCCTATTTGATGATGAA-3' for GSDME were synthesized and cloned into the pLKO.1-copGFP-PURO vector from GenePharma (Shanghai, China). All constructs were confirmed by DNA sequencing.

### Cell culture and transfection

HeLa, iBMDM, Huh7, SY5Y, HT-29, and MEF cells were grown in high-glucose-containing DMEM (Hyclone) containing 10% fetal bovine serum (Hyclone) and 100 units/ml penicillin-streptomycin (Hyclone). All cells were cultured at 37 °C in a 5% CO_2_ incubator (Thermo Fisher). To induce pyroptosis, HeLa cells were treated with 100 μM or 250 μM topotecan, 20 μM or 50 μM doxorubicin, or 50 μM or 100 μM etoposide. In comparison, Huh7 cells were treated by 2 μM, 5 μM or 10 μM sorafenib, or 10 μM, 20 μM, or 50 μM cisplatin. All of the chemotherapy drugs and Sp1 inhibitors above are from MedChemExpress. HT-29 and MEF cells were treated with 10 ng/mL TNF (Abcam), 100 nM Smac-mimetic (MedChemExpress), and 5 μM Z-VAD-FMK (Abcam) to induce necroptosis, and iBMDM cells were treated by 1 μg/mL LPS (Sigma), and 20 μM nigericin (MedChemExpress) to induce GSDMD-mediated pyroptosis. 200 μM peretinoin (MedChemExpress) or 200 nM plicamycin (MedChemExpress) were used to inhibit Sp1. Plasmids were transiently transfected into HeLa cells with Polyjet (SignaGen Laboratories) according to the manufacturer’s instructions.

### Lentivirus expression system

For lentiviral particle production, plko.1-copGFP-PURO plasmid containing scramble or target shRNA oligonucleotides, psPAX2, and VSVG plasmids were co-transfected into 293 T cells. The supernatants were harvested 48 h and 72 h post-transfection. To knockdown Sp1 in HT-29 and iBMDM, the indicated cells were infected with 2 ml of supernatant lentiviral particles for 48 h. Knockdown efficiency was assessed by GFP fluorescence and western blotting.

### Dual-luciferase reporter assay

HeLa cells were co-transfected with pGL-basic containing the candidate GSDME promoter truncation or mutation sequence and the control reporter RL-TK plasmids using Polyjet (SignaGen Laboratories). Cells supernatant was harvested to measure luciferase activity 48 h post-transfection by Dual-Luciferase Reporter Assay System kit (Beyotime) by SpectraMax M5 plate reader.

### EMSA assay

HeLa nuclear proteins were extracted using the nuclear and cytoplasmic protein extraction kit (Beyotime) according to the manufacturer’s protocol. FAM-labeled 30 bp dsDNA containing wild-type or mutated Sp1 binding sites were synthesized as probes. The indicated DNA binding reaction mixture was incubated on ice for 2 h, then separated on a 6% polyacrylamide gel and detected with a phosphor imager.

### Protein preparation and western blotting analysis

Cells were harvested and washed in PBS and lysed in ice-cold RIPA lysis buffer (50 mM Tris-HCl, pH 7.5, 150 mM NaCl, 1% Triton X-100 and 1 mM EDTA), supplemented with protease inhibitor cocktail (Roche). Lytic cells were centrifuged at 17,000 × g for 10 min at 4 °C. Proteins contained in the supernatant were separated by SDS-PAGE, and incubated with specific antibodies: anti-Sp1 (Abcam, ab231778), anti-GSDME (Abcam, ab215191), anti-GSDMD (Abcam, ab209845), anti-RIP3(Santa Cruz, sc-374639), anti-MLKL (Abcam, ab172868), anti-STAT3 (Abcam, ab68153), anti-caspase 3 (Cell Signaling Technology, 14220), anti-caspase 8 (Cell Signaling Technology, 4790), anti-NINJ1 (Santa Cruz, sc-136295), and anti-β-actin (Proteintech, 66009-1-Ig).

### Reverse transcription-PCR (RT-PCR)

The total RNA was extracted with TRIzol from indicated cell lines according to the manufacturer’s instructions (Invitrogen), and was reverse-transcribed into cDNA using the PrimeScript RT Master Mix kit (Takara). Specific primers used for RT-PCR assays were 5'-CCACAGTTCCAGACCGTTGA-3', 5'-CTGCTGGAGTAGGTTTTGGCA-3' for Sp1; 5'-GGTCTTTCGAGAGTTTGCATTCA-3', 5'-AGATGTCACTCAAAGCTGTCTGT-3' for GSDME; 5'-GCTGCTCATCTTCCTTGTCAAGTA-3', 5'-TGAAGATGTTGACTACCACGATGA-3' for NINJ1; 5'-TCAACAAGAGCCTGCTGAAGATA-3', 5'-GGAGAGTCCGAGATTGTCATTAC-3' for caspase 8; 5'-GGAAGCGAATCAATGGACTCTGG-3', 5'-GCATCGACATCTGTACCAGACC-3' for caspase 3; 5'-TGTTACCAACTGGGACGACA-3', 5'-CTGGGTCATCTTTTCACGGT-3' for β-actin. The gene expression levels were normalized to those of β-actin.

### Cytotoxicity and cell viability assays

Cell viability was measured after chemotherapy drug stimulation using the Cell Counting Kit-8 (APExBIO), while cytotoxicity was determined by the lactate dehydrogenase (LDH) release using LDH Cytotoxicity Assay Kit (Beyotime), according to the manufacturer’s instructions. The absorbance was measured on the SpectraMax M5 plate reader.

### Statistical analysis

Each experiment was performed at least three times. All experiment data were analyzed using GraphPad Prism 9.0 (GraphPad Software Inc. USA) and were presented as the mean ± SD. Statistical analysis was performed using Student’s t-test, one-way ANOVA, or two-way ANOVA. A value of *P* < 0.05 was considered statistically significant.

### Supplementary information

Supplementary file

aj-checklist

Original Data File

Authorship confirmation letter
