# Supplementary material for: Transcription factor Sp1 transcriptionally enhances GSDME expression for pyroptosis
Source: Cell Death Dis. 2024 Jan 18;15(1):66. doi: 10.1038/s41419-024-06455-6 (PMC10796635; doi:10.1038/s41419-024-06455-6)
Supplement: Supplementary file 1 — Supplementary file [file 41419_2024_6455_MOESM1_ESM.docx]

**Transcription factor Sp1 transcriptionally enhances GSDME expression for pyroptosis**

Jiasong Pan^1^, Yuanyuan Li^1^, Wenqing Gao^1^, Qizhou Jiang^2^, Lu Geng^1^, Jin Ding^3^, Suhua Li^2,*^, Jixi Li^1,3,^*

^1^Department of Neurology, Huashan Hospital, State Key Laboratory of Genetic Engineering and School of Life Sciences, Fudan University, Shanghai, 200438, China

^2^Division of Natural Science, Duke Kunshan University, Jiangsu, 215316, China

^3^Clinical Cancer Institute, Center for Translational Medicine, Naval Medical University, Shanghai 200433, China.

*To whom correspondence should be addressed. Email: [lijixi@fudan.edu.cn](mailto:lijixi@fudan.edu.cn) or [suhua.li@dukekunshan.edu.cn](mailto:suhua.li@dukekunshan.edu.cn)

**Supplementary Figures**

**
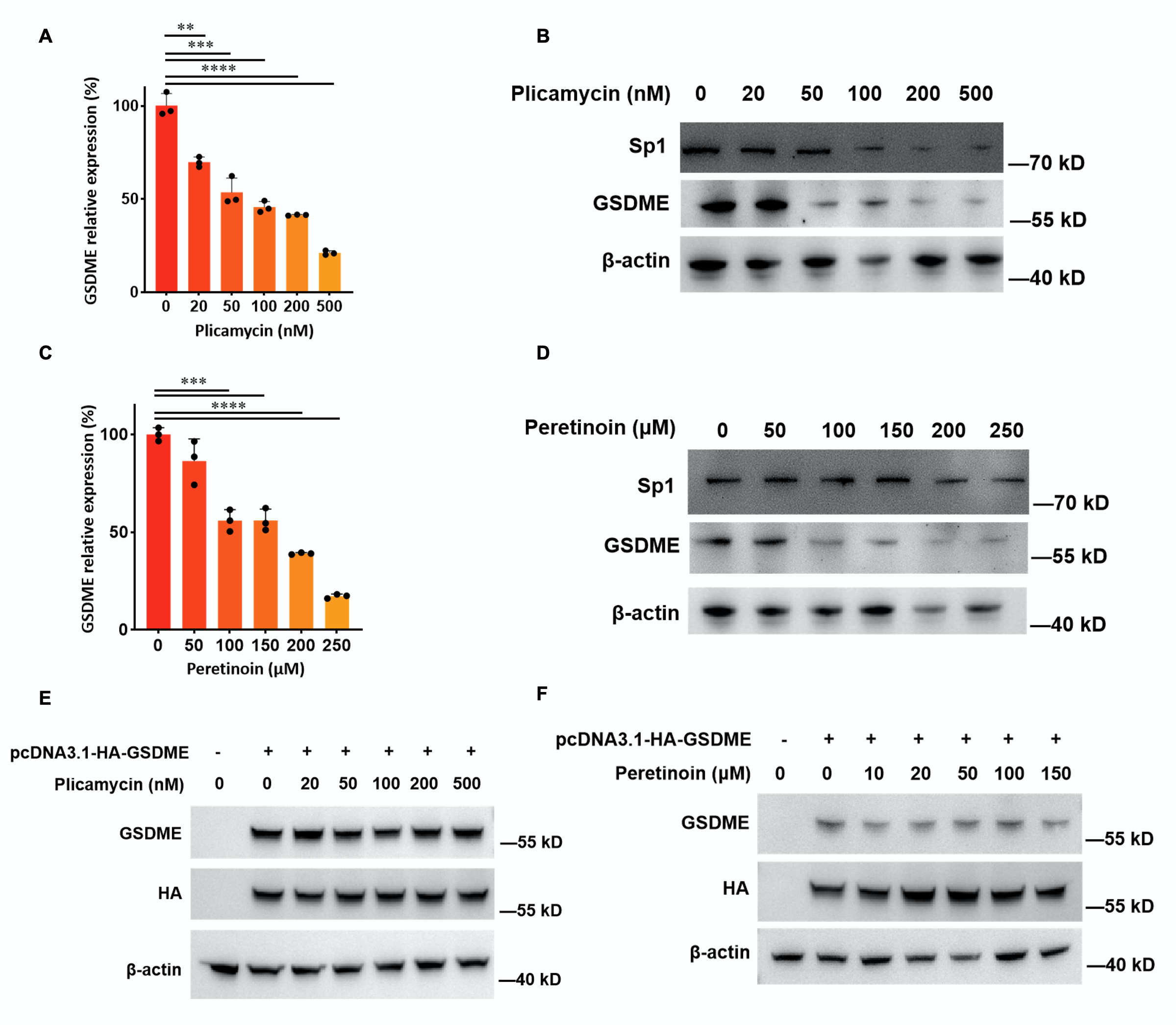
**

**Figure S1.** **Sp1 regulates GSDME expression in Huh7 cells.**

(**A and C**) qPCR analysis result of GSDME mRNA levels in Huh7 cells treated with the indicated amounts of plicamycin or peretinoin, which is normalized by β-actin. (**B and D**) Huh7 cells treated with the indicated amounts of plicamycin or peretinoin were harvested and lysed in the RIPA lysis buffer. Western blotting analysis of lysates from the cells shows the protein expression levels of Sp1, GSDME, and β-actin. (**E and F**) HEK293T cells transfected with pcDNA3.1-HA-GSDME plasmid and treated by the indicated amounts of plicamycin or peretinoin were harvested and lysed in the RIPA lysis buffer. Western blotting analysis of lysates from the cells shows the protein expression levels of HA, GSDME, and β-actin.

**
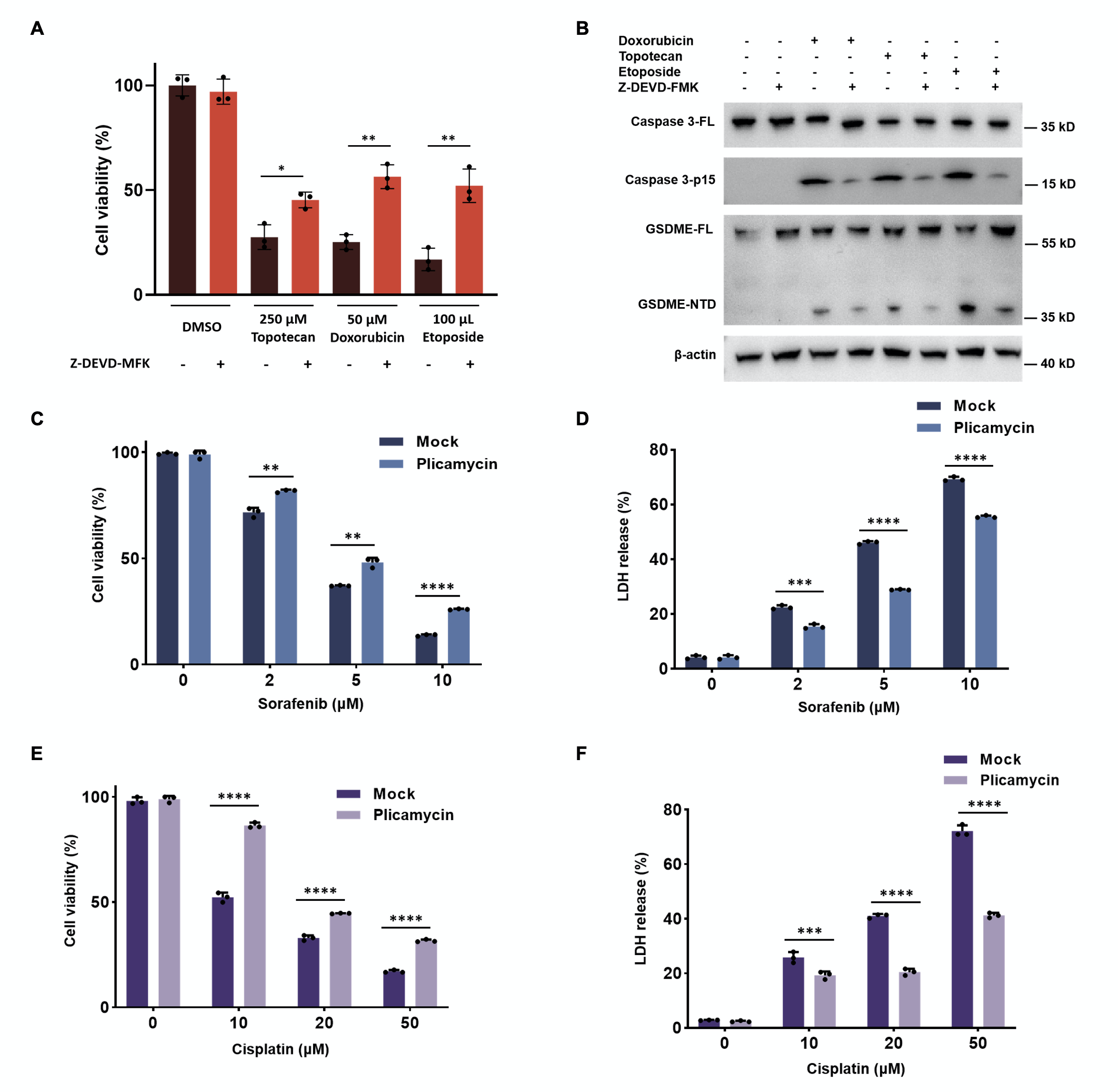
Figure S2.** **Sp1 modulates GSDME-mediated pyroptosis in Huh7 cells.**

(**A and B**) HeLa cells were treated with Z-DEVD-FMK for 24 h, followed by chemotherapy drugs topotecan, doxorubicin, or etoposide for another 24 h. CCK8 analysis was performed to measure cell viability. Western blotting analysis of lysates from the cells shows the protein expression levels of FL/cleaved-caspase 3, FL/cleaved-GSDME, and β-actin. (**C, D, E, and F**) Huh7 cells were treated with plicamycin for 24 h, followed by indicated chemotherapy drugs for another 24 h. Cell viability and LDH release were determined, respectively.

**
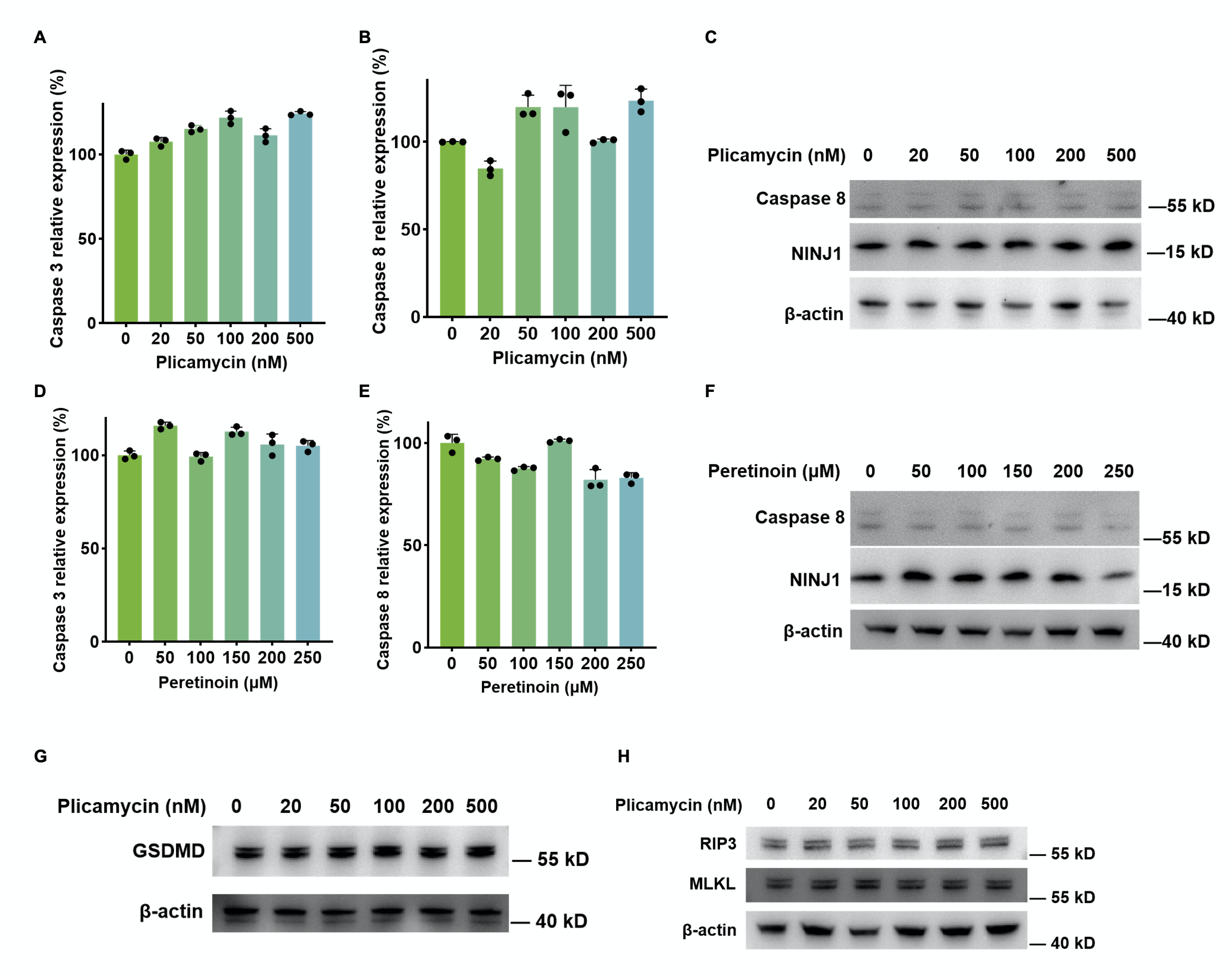
Figure S3.** **Sp1 does not affect other pyroptotic gene expression in SY5Y cells.**

(**A, B, D, and E**) qPCR analysis results of caspase 3 and caspase 8 mRNA levels in the neuroblastoma SY5Y cells treated by the indicated amounts of plicamycin or peretinoin, normalized by β-actin. (**C and F**) SY5Y cells treated with the indicated amount of plicamycin or peretinoin were harvested and lysed in the RIPA lysis buffer. Western blotting analysis of lysates from the cells show the protein expression levels of NINJ1, caspase 8, and β-actin. (**G**) Western blotting analysis of lysates from iBMDM cells treated with the indicated amount of plicamycin shows the protein expression levels of GSDMD and β-actin. (**H**) Western blotting analysis of lysates from HT-29 cells treated with the indicated amount of plicamycin shows human RIP3, MLKL, and β-actin protein expression levels.
