# Supplementary material for: Transcription factor Sp1 transcriptionally enhances GSDME expression for pyroptosis
Source: Cell Death Dis. 2024 Jan 18;15(1):66. doi: 10.1038/s41419-024-06455-6 (PMC10796635; doi:10.1038/s41419-024-06455-6)
Supplement: Supplementary file 3 — Original Data File [file 41419_2024_6455_MOESM3_ESM.pptx]

## Slide 1
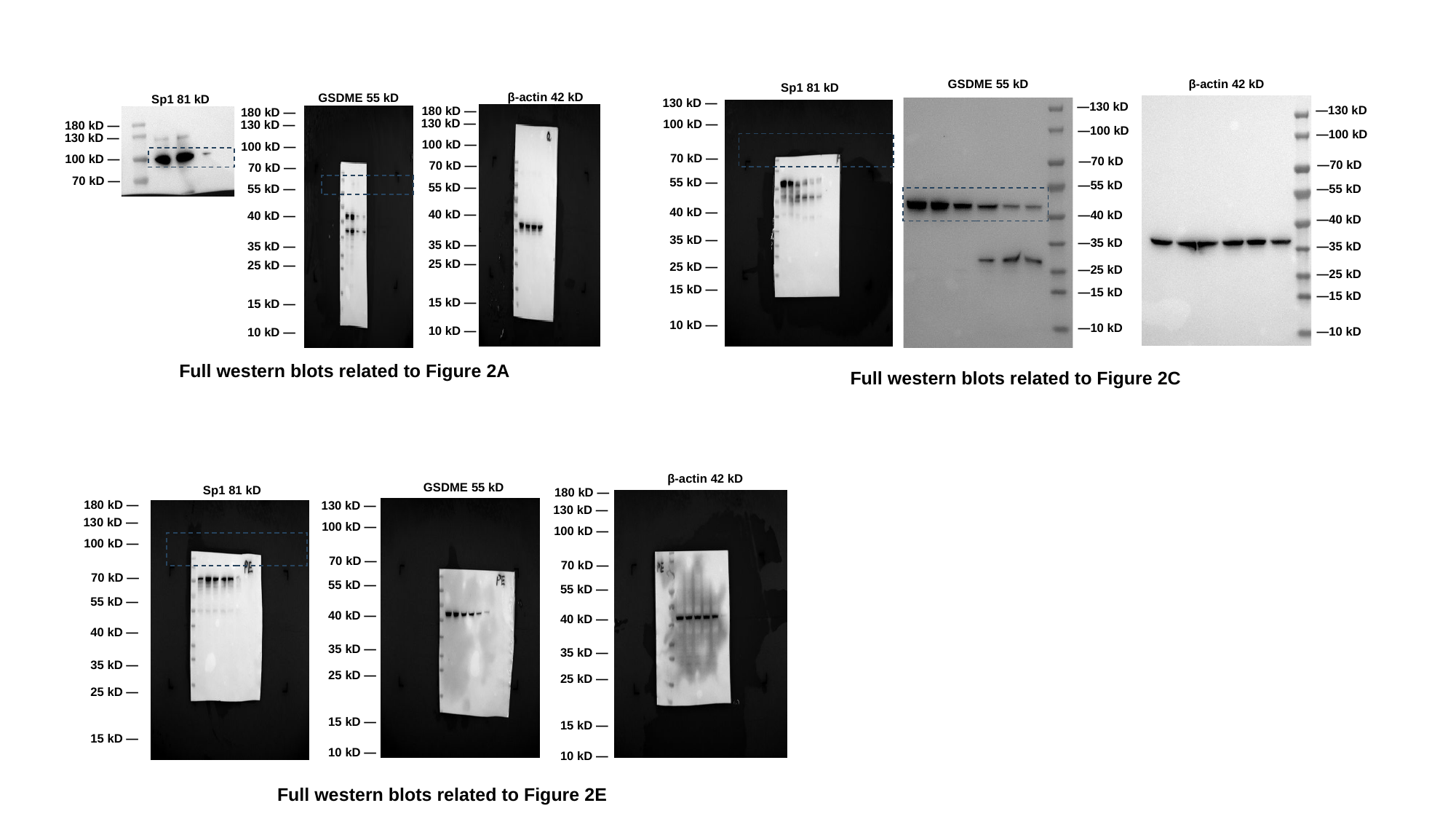

β-actin 42 kD
—130 kD
—100 kD
—70 kD
—55 kD
—40 kD
—35 kD
—25 kD
—15 kD
—10 kD
GSDME 55 kD
Sp1 81 kD
130 kD —
100 kD —
70 kD —
55 kD —
40 kD —
35 kD —
25 kD —
15 kD —
10 kD —
—130 kD
—100 kD
—70 kD
—55 kD
—40 kD
—35 kD
—25 kD
—15 kD
—10 kD
Full western blots related to Figure 2C
β-actin 42 kD
180 kD —
130 kD —
100 kD —
70 kD —
55 kD —
40 kD —
35 kD —
25 kD —
15 kD —
10 kD —
GSDME 55 kD
Sp1 81 kD
180 kD —
130 kD —
100 kD —
70 kD —
180 kD —
130 kD —
100 kD —
70 kD —
55 kD —
40 kD —
35 kD —
25 kD —
15 kD —
10 kD —
Full western blots related to Figure 2A
β-actin 42 kD
GSDME 55 kD
Sp1 81 kD
180 kD —
180 kD —
130 kD —
100 kD —
70 kD —
55 kD —
40 kD —
35 kD —
25 kD —
15 kD —
130 kD —
100 kD —
70 kD —
55 kD —
40 kD —
35 kD —
25 kD —
15 kD —
10 kD —
130 kD —
100 kD —
70 kD —
55 kD —
40 kD —
35 kD —
25 kD —
15 kD —
10 kD —
Full western blots related to Figure 2E

## Slide 2
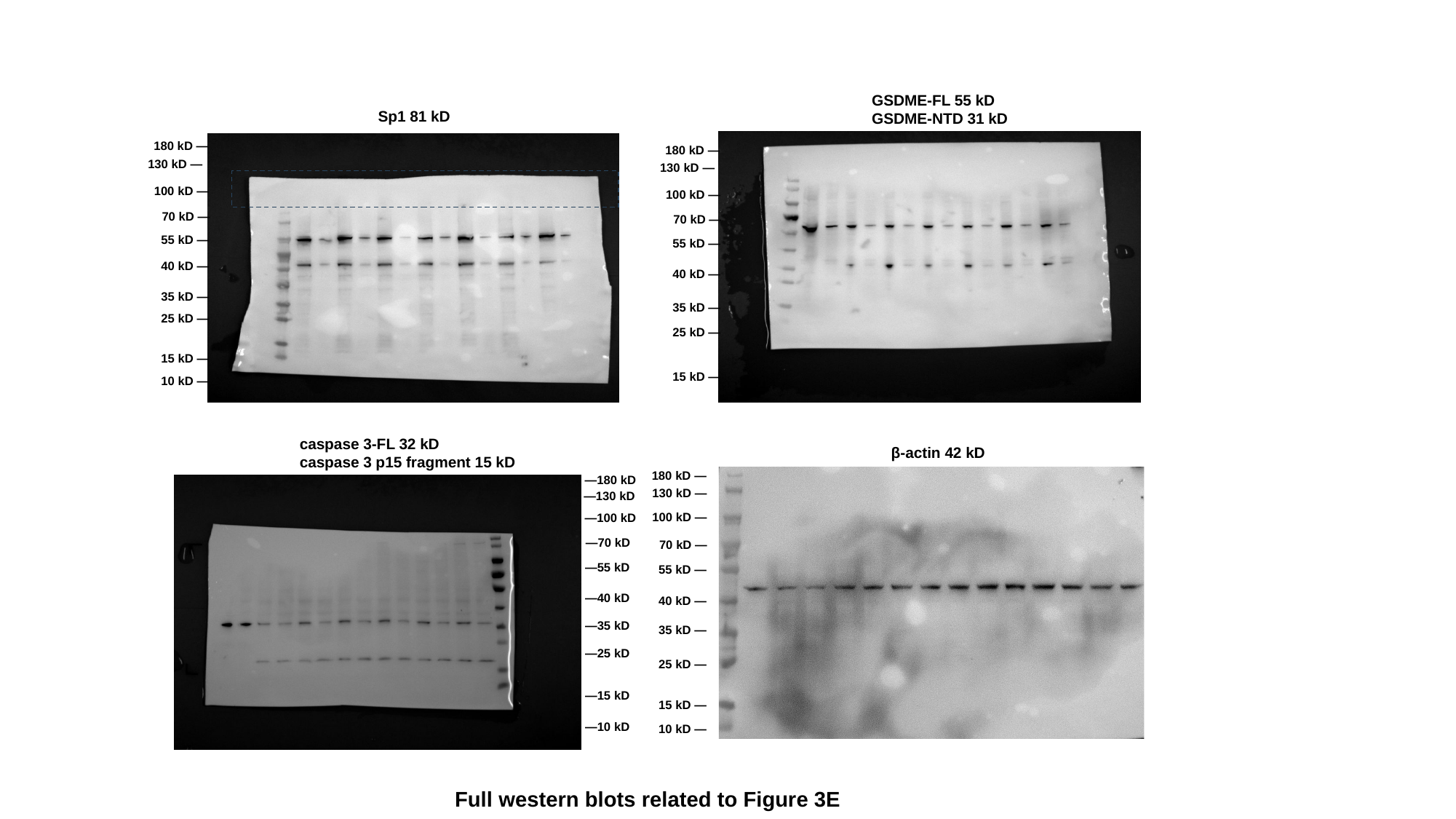

GSDME-FL 55 kD
GSDME-NTD 31 kD
180 kD —
130 kD —
100 kD —
70 kD —
55 kD —
40 kD —
35 kD —
25 kD —
15 kD —
Sp1 81 kD
180 kD —
130 kD —
100 kD —
70 kD —
55 kD —
40 kD —
35 kD —
25 kD —
15 kD —
10 kD —
caspase 3-FL 32 kD
caspase 3 p15 fragment 15 kD
β-actin 42 kD
180 kD —
130 kD —
100 kD —
70 kD —
55 kD —
40 kD —
35 kD —
25 kD —
15 kD —
10 kD —
—180 kD
—130 kD
—100 kD
—70 kD
—55 kD
—40 kD
—35 kD
—25 kD
—15 kD
—10 kD
Full western blots related to Figure 3E

## Slide 3
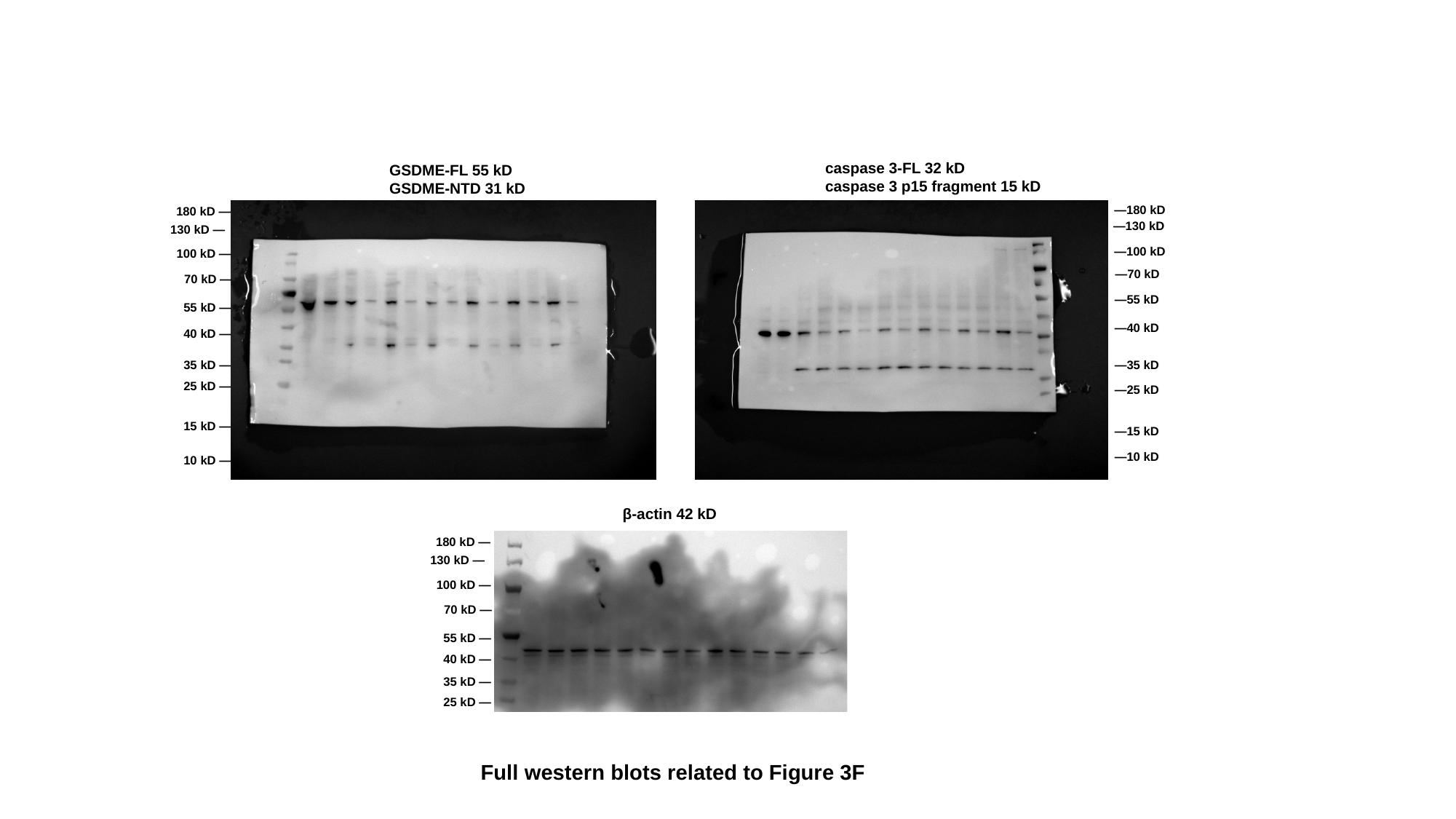

caspase 3-FL 32 kD
caspase 3 p15 fragment 15 kD
GSDME-FL 55 kD
GSDME-NTD 31 kD
—180 kD
—130 kD
—100 kD
—70 kD
—55 kD
—40 kD
—35 kD
—25 kD
—15 kD
—10 kD
180 kD —
130 kD —
100 kD —
70 kD —
55 kD —
40 kD —
35 kD —
25 kD —
15 kD —
10 kD —
β-actin 42 kD
180 kD —
130 kD —
100 kD —
70 kD —
55 kD —
40 kD —
35 kD —
25 kD —
Full western blots related to Figure 3F

## Slide 4
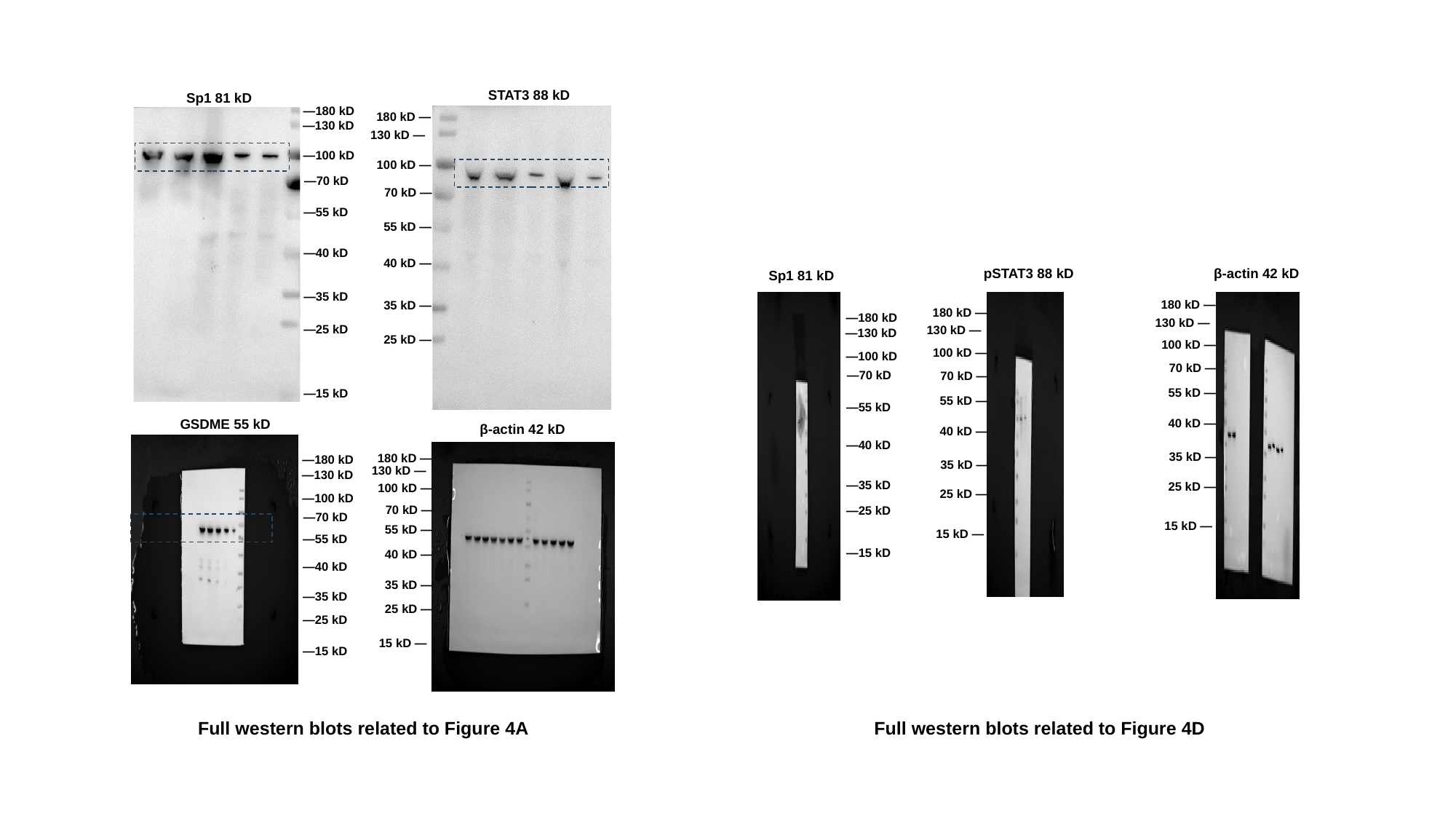

STAT3 88 kD
Sp1 81 kD
—180 kD
—130 kD
—100 kD
—70 kD
—55 kD
—40 kD
—35 kD
—25 kD
—15 kD
180 kD —
130 kD —
100 kD —
70 kD —
55 kD —
40 kD —
35 kD —
25 kD —
GSDME 55 kD
β-actin 42 kD
180 kD —
130 kD —
100 kD —
70 kD —
55 kD —
40 kD —
35 kD —
25 kD —
—180 kD
—130 kD
—100 kD
—70 kD
—55 kD
—40 kD
—35 kD
—25 kD
—15 kD
15 kD —
pSTAT3 88 kD
β-actin 42 kD
Sp1 81 kD
180 kD —
130 kD —
100 kD —
70 kD —
55 kD —
40 kD —
35 kD —
25 kD —
15 kD —
180 kD —
130 kD —
100 kD —
70 kD —
55 kD —
40 kD —
35 kD —
25 kD —
15 kD —
—180 kD
—130 kD
—100 kD
—70 kD
—55 kD
—40 kD
—35 kD
—25 kD
—15 kD
Full western blots related to Figure 4A
Full western blots related to Figure 4D

## Slide 5
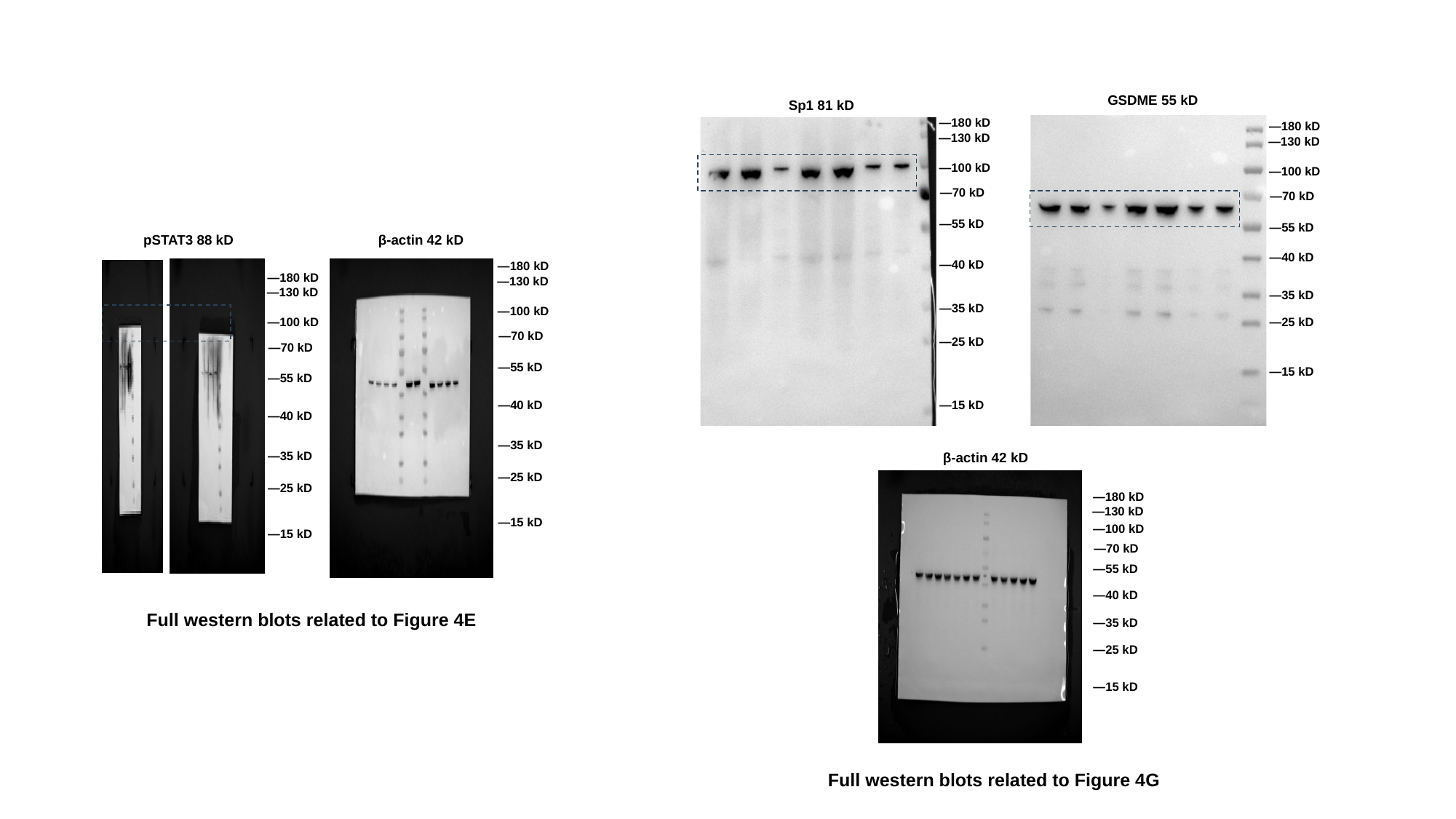

GSDME 55 kD
Sp1 81 kD
—180 kD
—130 kD
—100 kD
—70 kD
—55 kD
—40 kD
—35 kD
—25 kD
—15 kD
—180 kD
—130 kD
—100 kD
—70 kD
—55 kD
—40 kD
—35 kD
—25 kD
—15 kD
β-actin 42 kD
—180 kD
—130 kD
—100 kD
—70 kD
—55 kD
—40 kD
—35 kD
—25 kD
—15 kD
Full western blots related to Figure 4G
pSTAT3 88 kD
β-actin 42 kD
—180 kD
—130 kD
—100 kD
—70 kD
—55 kD
—40 kD
—35 kD
—25 kD
—15 kD
—180 kD
—130 kD
—100 kD
—70 kD
—55 kD
—40 kD
—35 kD
—25 kD
—15 kD
Full western blots related to Figure 4E

## Slide 6
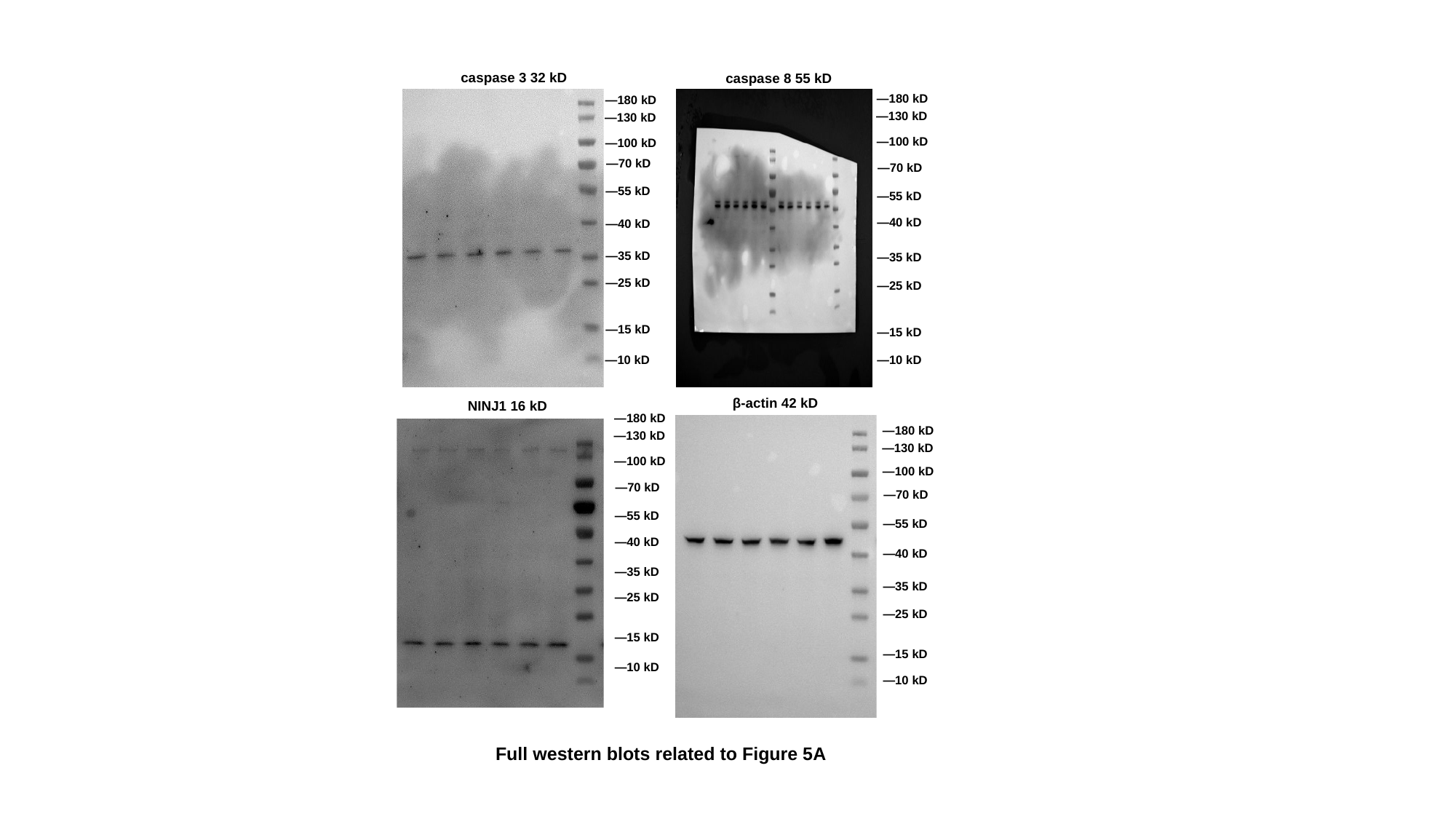

caspase 3 32 kD
caspase 8 55 kD
—180 kD
—130 kD
—100 kD
—70 kD
—55 kD
—40 kD
—35 kD
—25 kD
—15 kD
—10 kD
—180 kD
—130 kD
—100 kD
—70 kD
—55 kD
—40 kD
—35 kD
—25 kD
—15 kD
—10 kD
β-actin 42 kD
NINJ1 16 kD
—180 kD
—130 kD
—100 kD
—70 kD
—55 kD
—40 kD
—35 kD
—25 kD
—15 kD
—10 kD
—180 kD
—130 kD
—100 kD
—70 kD
—55 kD
—40 kD
—35 kD
—25 kD
—15 kD
—10 kD
Full western blots related to Figure 5A

## Slide 7
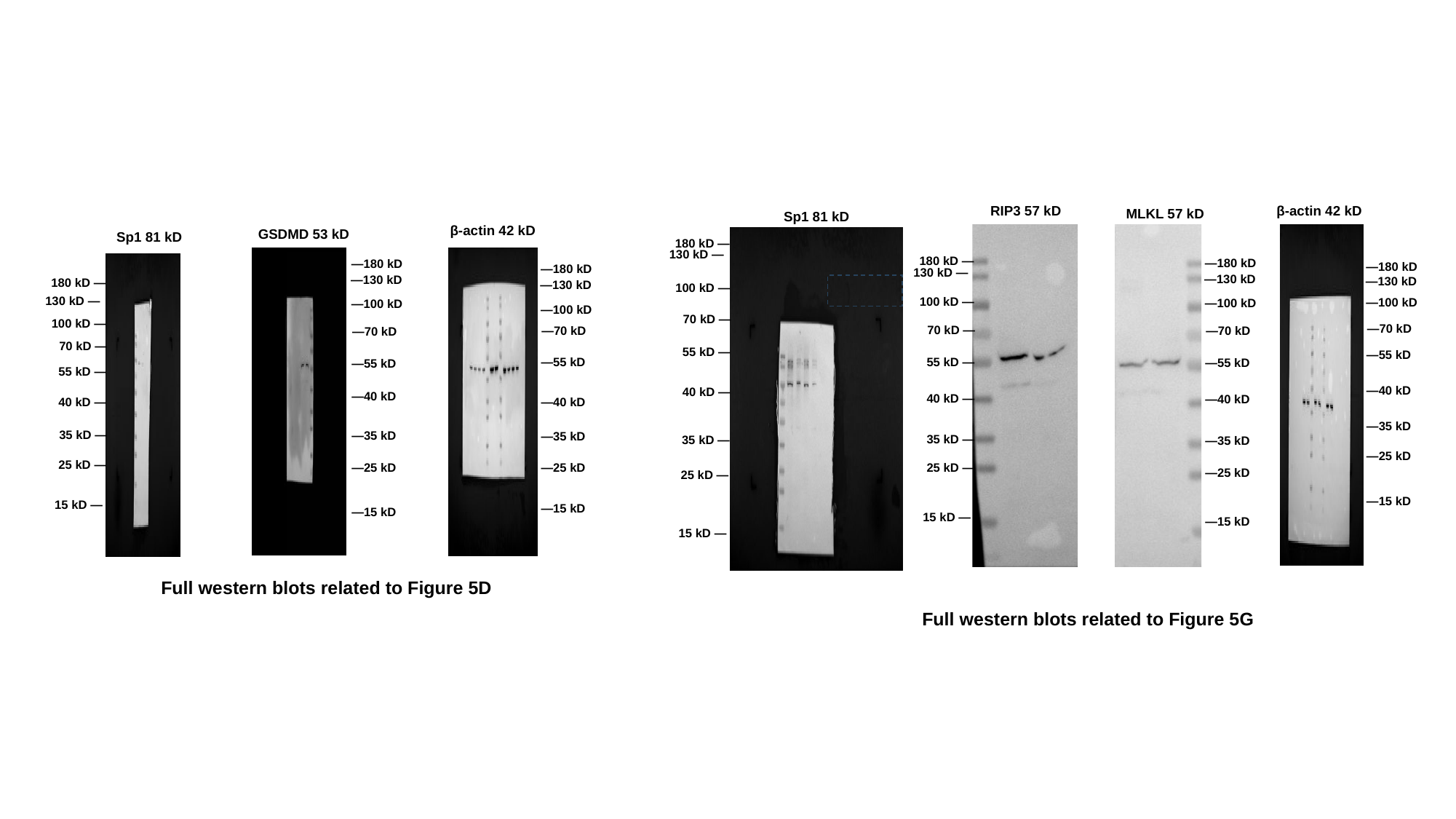

β-actin 42 kD
RIP3 57 kD
MLKL 57 kD
Sp1 81 kD
180 kD —
130 kD —
100 kD —
70 kD —
55 kD —
40 kD —
35 kD —
25 kD —
15 kD —
180 kD —
130 kD —
100 kD —
70 kD —
55 kD —
40 kD —
35 kD —
25 kD —
15 kD —
—180 kD
—130 kD
—100 kD
—70 kD
—55 kD
—40 kD
—35 kD
—25 kD
—15 kD
—180 kD
—130 kD
—100 kD
—70 kD
—55 kD
—40 kD
—35 kD
—25 kD
—15 kD
Full western blots related to Figure 5G
β-actin 42 kD
GSDMD 53 kD
Sp1 81 kD
—180 kD
—130 kD
—100 kD
—70 kD
—55 kD
—40 kD
—35 kD
—25 kD
—15 kD
—180 kD
—130 kD
—100 kD
—70 kD
—55 kD
—40 kD
—35 kD
—25 kD
—15 kD
180 kD —
130 kD —
100 kD —
70 kD —
55 kD —
40 kD —
35 kD —
25 kD —
15 kD —
Full western blots related to Figure 5D

## Slide 8
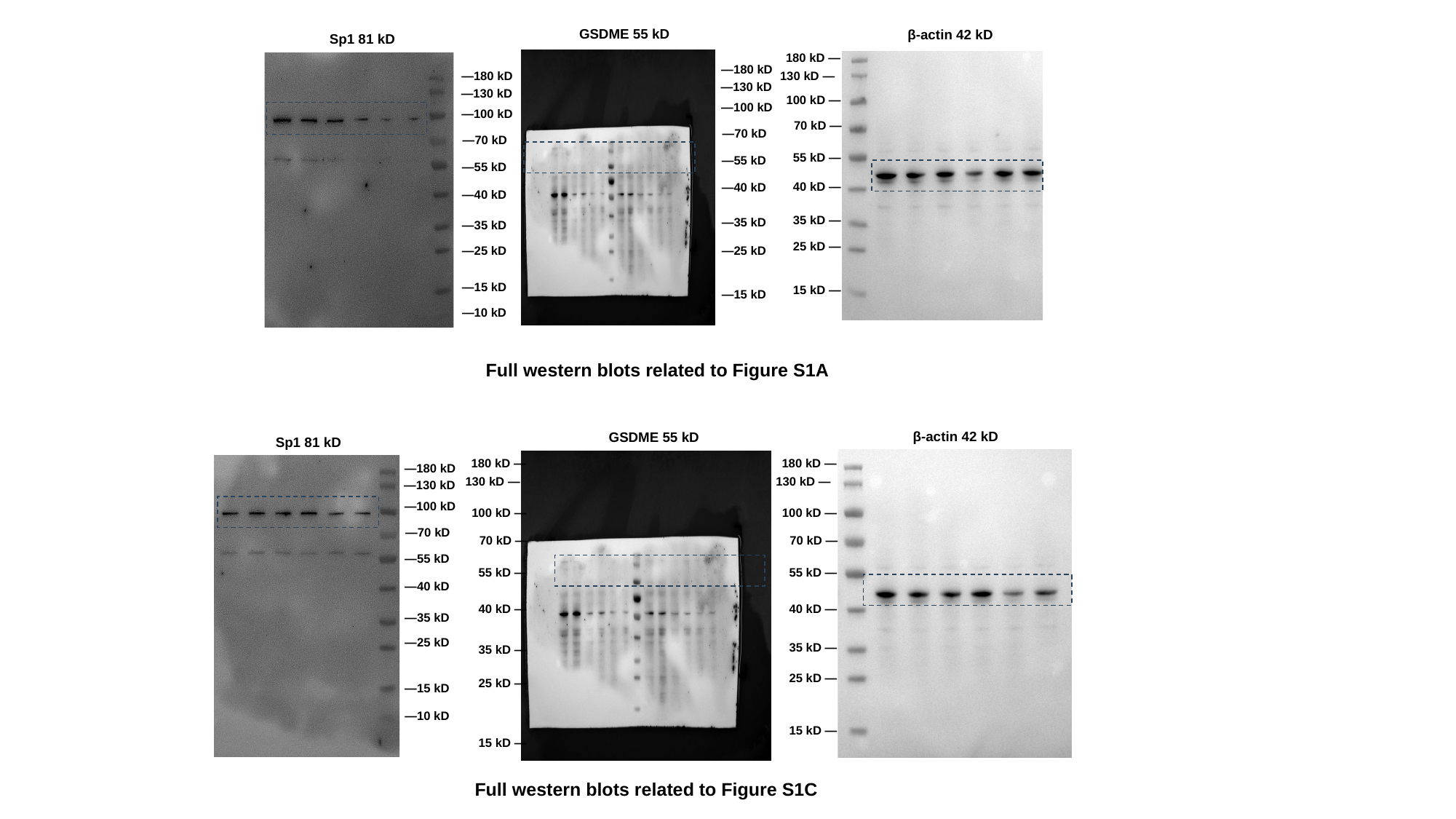

GSDME 55 kD
β-actin 42 kD
Sp1 81 kD
180 kD —
130 kD —
100 kD —
70 kD —
55 kD —
40 kD —
35 kD —
25 kD —
15 kD —
—180 kD
—130 kD
—100 kD
—70 kD
—55 kD
—40 kD
—35 kD
—25 kD
—15 kD
—180 kD
—130 kD
—100 kD
—70 kD
—55 kD
—40 kD
—35 kD
—25 kD
—15 kD
—10 kD
Full western blots related to Figure S1A
β-actin 42 kD
GSDME 55 kD
Sp1 81 kD
180 kD —
130 kD —
100 kD —
70 kD —
55 kD —
40 kD —
35 kD —
25 kD —
15 kD —
180 kD —
130 kD —
100 kD —
70 kD —
55 kD —
40 kD —
35 kD —
25 kD —
15 kD —
—180 kD
—130 kD
—100 kD
—70 kD
—55 kD
—40 kD
—35 kD
—25 kD
—15 kD
—10 kD
Full western blots related to Figure S1C

## Slide 9
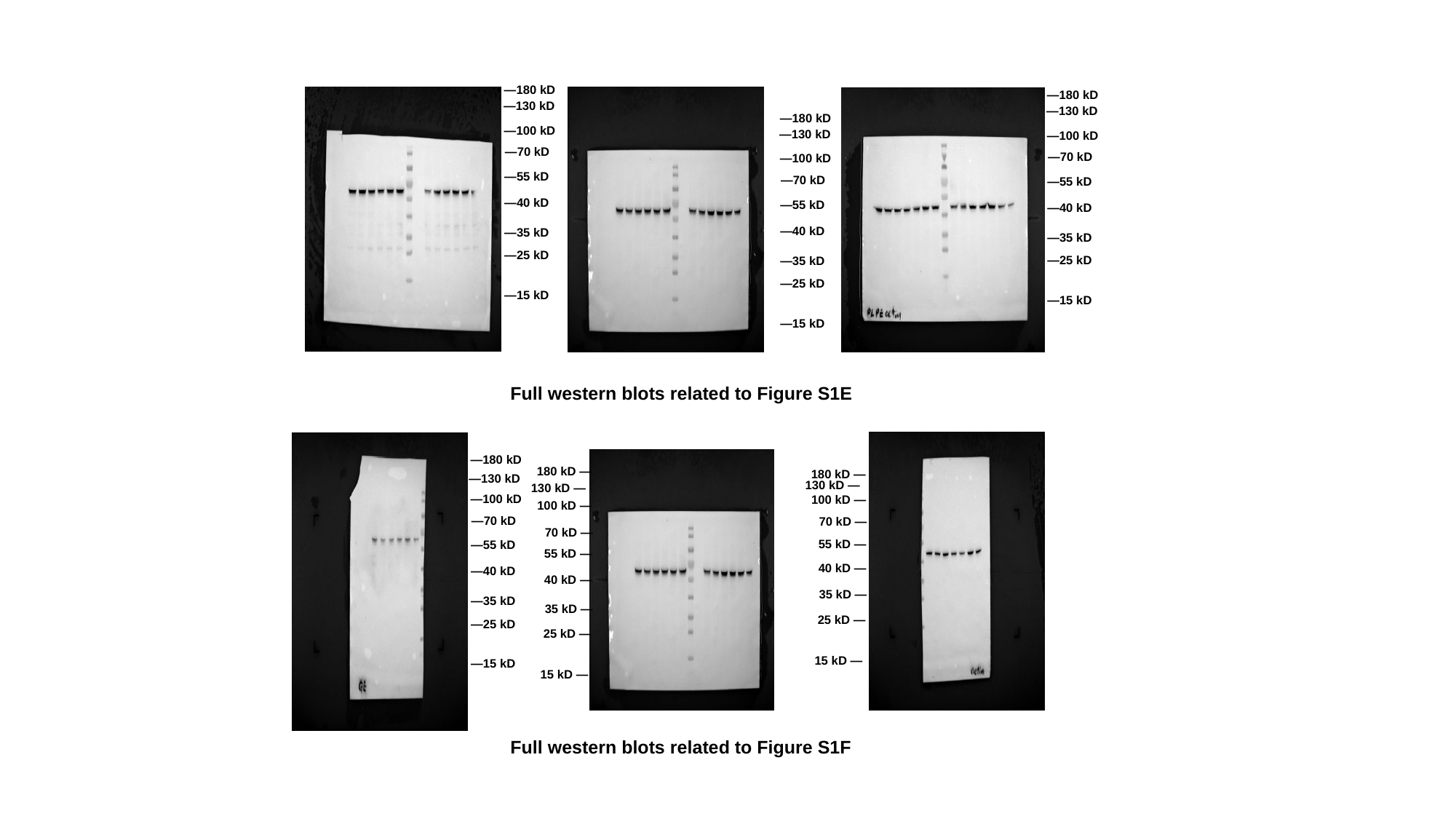

—180 kD
—130 kD
—100 kD
—70 kD
—55 kD
—40 kD
—35 kD
—25 kD
—15 kD
—180 kD
—130 kD
—100 kD
—70 kD
—55 kD
—40 kD
—35 kD
—25 kD
—15 kD
—180 kD
—130 kD
—100 kD
—70 kD
—55 kD
—40 kD
—35 kD
—25 kD
—15 kD
Full western blots related to Figure S1E
—180 kD
—130 kD
—100 kD
—70 kD
—55 kD
—40 kD
—35 kD
—25 kD
—15 kD
180 kD —
130 kD —
100 kD —
70 kD —
55 kD —
40 kD —
35 kD —
25 kD —
15 kD —
180 kD —
130 kD —
100 kD —
70 kD —
55 kD —
40 kD —
35 kD —
25 kD —
15 kD —
Full western blots related to Figure S1F

## Slide 10
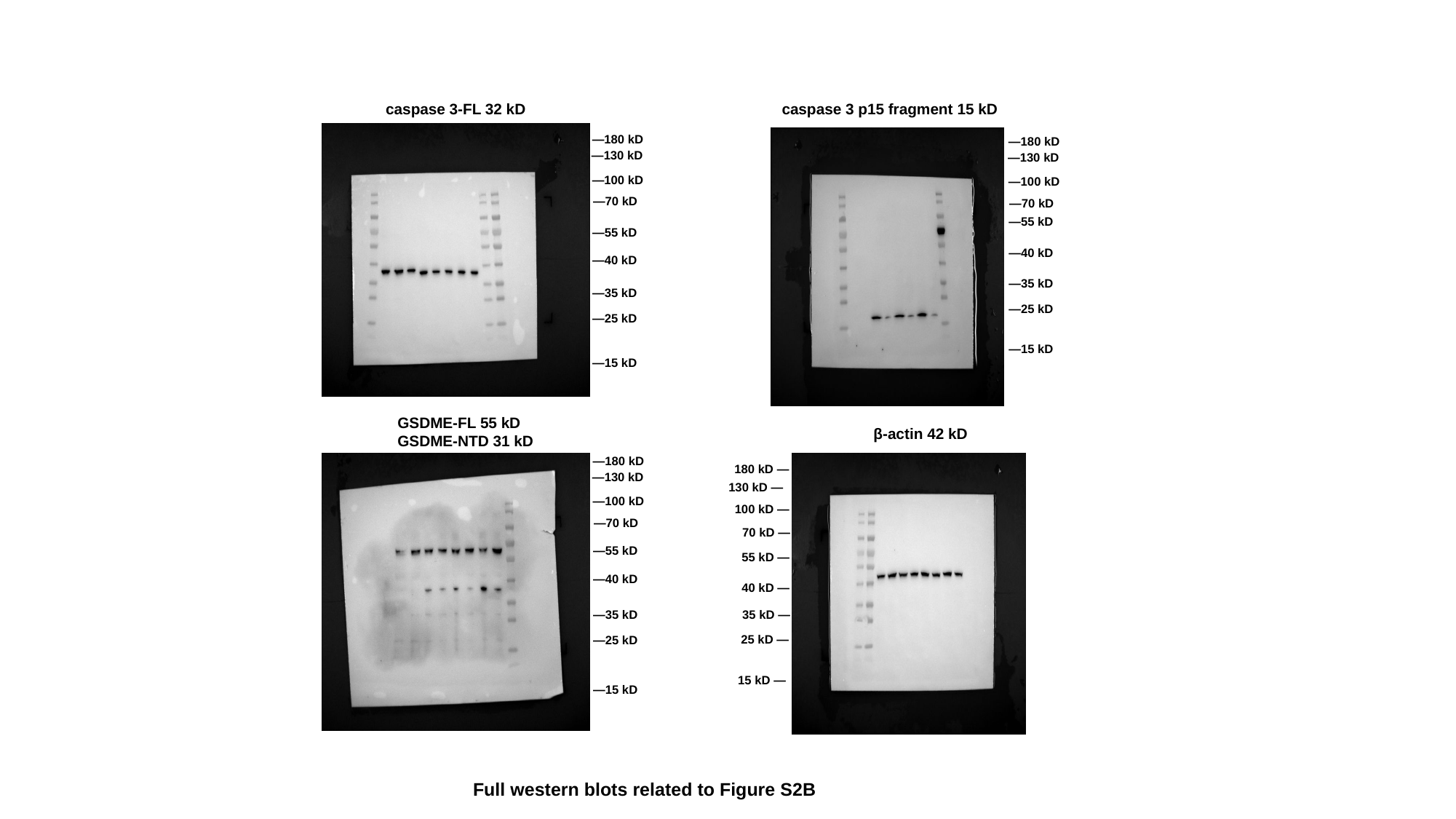

caspase 3-FL 32 kD
caspase 3 p15 fragment 15 kD
—180 kD
—130 kD
—100 kD
—70 kD
—55 kD
—40 kD
—35 kD
—25 kD
—15 kD
—180 kD
—130 kD
—100 kD
—70 kD
—55 kD
—40 kD
—35 kD
—25 kD
—15 kD
GSDME-FL 55 kD
GSDME-NTD 31 kD
β-actin 42 kD
—180 kD
—130 kD
—100 kD
—70 kD
—55 kD
—40 kD
—35 kD
—25 kD
—15 kD
180 kD —
130 kD —
100 kD —
70 kD —
55 kD —
40 kD —
35 kD —
25 kD —
15 kD —
Full western blots related to Figure S2B

## Slide 11
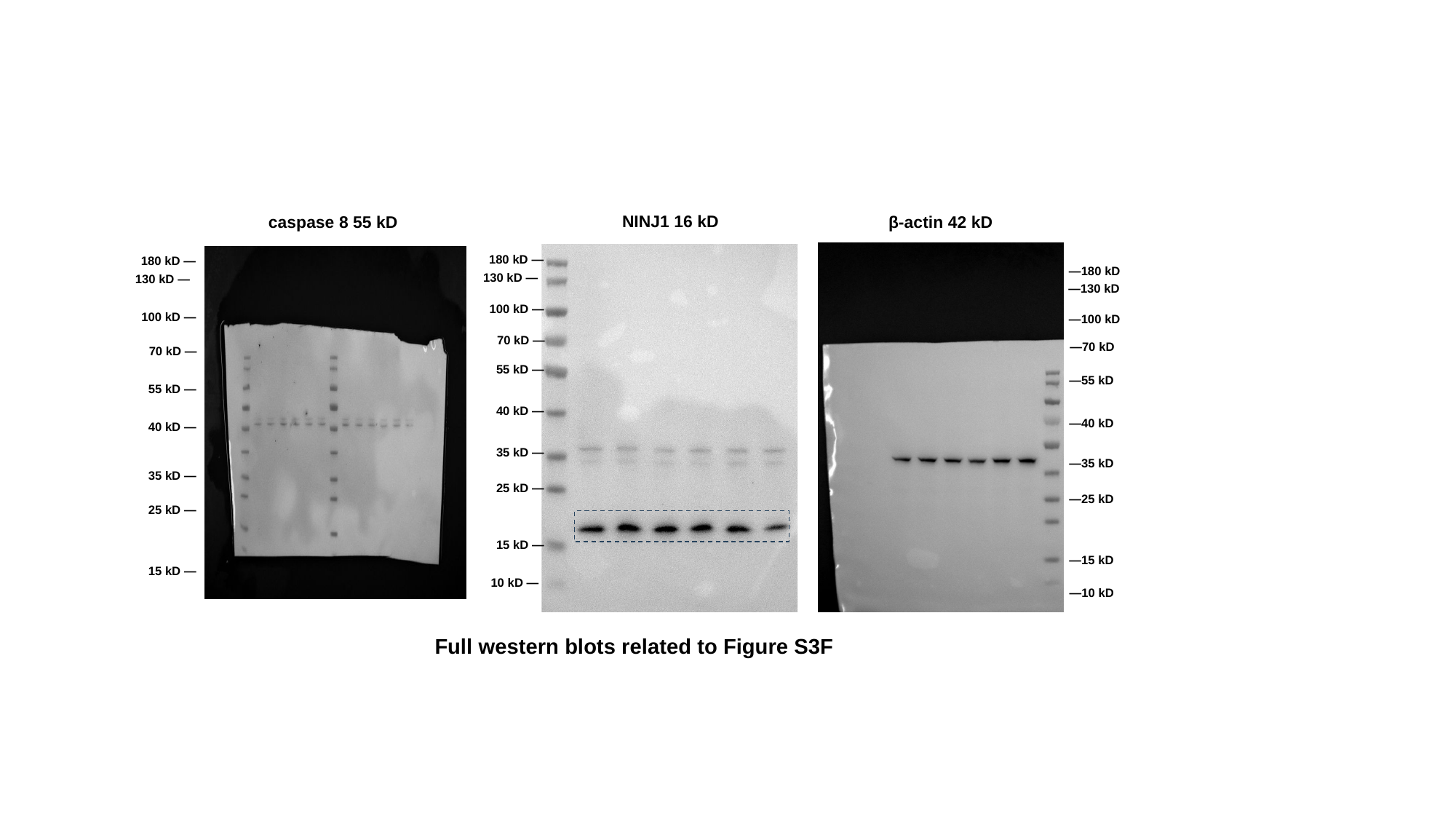

NINJ1 16 kD
caspase 8 55 kD
β-actin 42 kD
180 kD —
130 kD —
100 kD —
70 kD —
55 kD —
40 kD —
35 kD —
25 kD —
15 kD —
10 kD —
180 kD —
130 kD —
100 kD —
70 kD —
55 kD —
40 kD —
35 kD —
25 kD —
15 kD —
—180 kD
—130 kD
—100 kD
—70 kD
—55 kD
—40 kD
—35 kD
—25 kD
—15 kD
—10 kD
Full western blots related to Figure S3F

## Slide 12
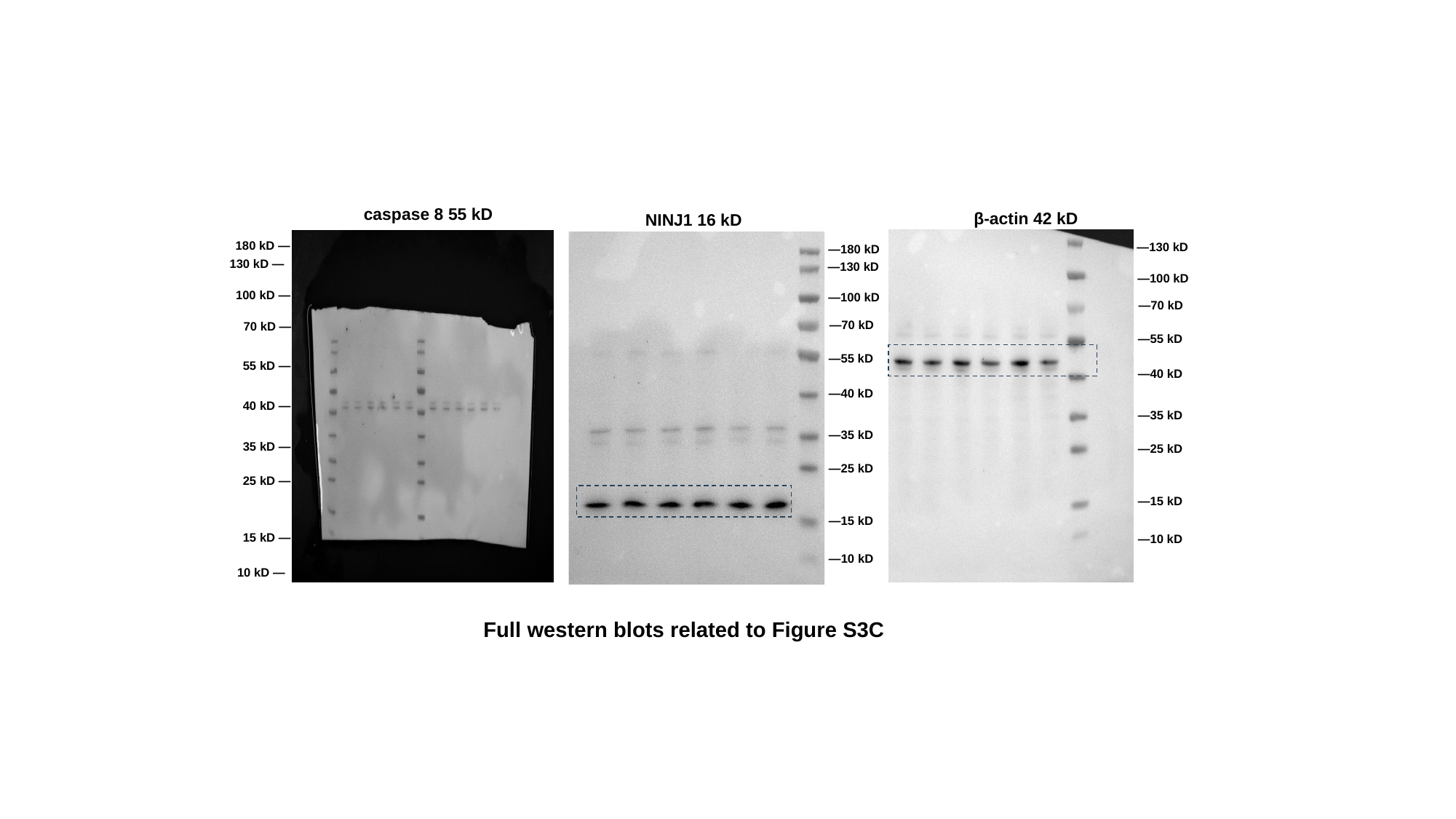

caspase 8 55 kD
β-actin 42 kD
NINJ1 16 kD
180 kD —
130 kD —
100 kD —
70 kD —
55 kD —
40 kD —
35 kD —
25 kD —
15 kD —
10 kD —
—130 kD
—100 kD
—70 kD
—55 kD
—40 kD
—35 kD
—25 kD
—15 kD
—10 kD
—180 kD
—130 kD
—100 kD
—70 kD
—55 kD
—40 kD
—35 kD
—25 kD
—15 kD
—10 kD
Full western blots related to Figure S3C

## Slide 13
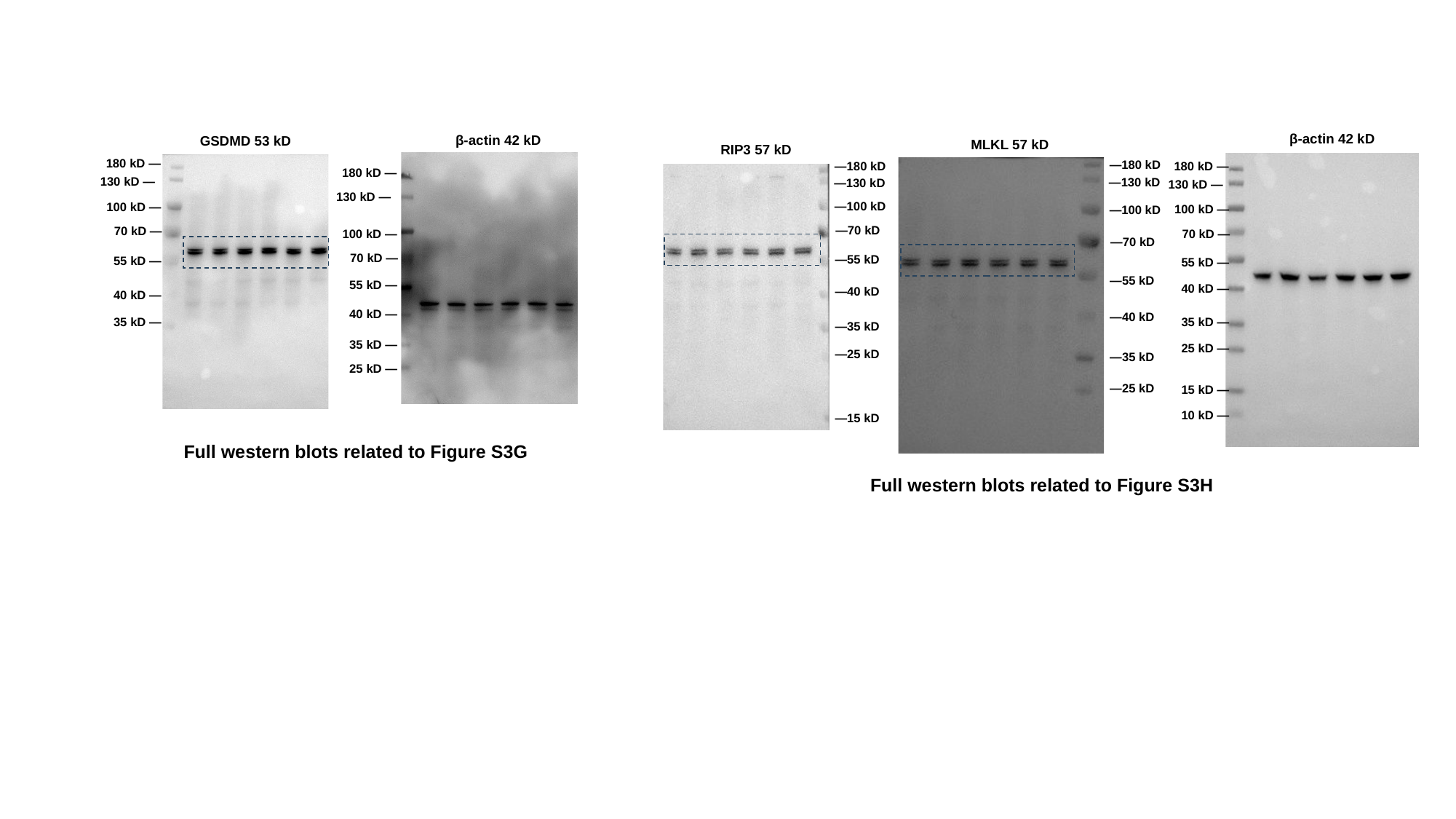

β-actin 42 kD
MLKL 57 kD
RIP3 57 kD
—180 kD
—130 kD
—100 kD
—70 kD
—55 kD
—40 kD
—35 kD
—25 kD
—180 kD
—130 kD
—100 kD
—70 kD
—55 kD
—40 kD
—35 kD
—25 kD
—15 kD
180 kD —
130 kD —
100 kD —
70 kD —
55 kD —
40 kD —
35 kD —
25 kD —
15 kD —
10 kD —
Full western blots related to Figure S3H
β-actin 42 kD
GSDMD 53 kD
180 kD —
130 kD —
100 kD —
70 kD —
55 kD —
40 kD —
35 kD —
180 kD —
130 kD —
100 kD —
70 kD —
55 kD —
40 kD —
35 kD —
25 kD —
Full western blots related to Figure S3G
