# Supplementary material for: Transcription factor Sp1 transcriptionally enhances GSDME expression for pyroptosis
Source: Cell Death Dis. 2024 Jan 18;15(1):66. doi: 10.1038/s41419-024-06455-6 (PMC10796635; doi:10.1038/s41419-024-06455-6)
Supplement: Supplementary file 4 — Authorship confirmation letter [file 41419_2024_6455_MOESM4_ESM.pdf]

Re: Authorships of Cell Death & Disease paper (CDDIS-23-3415RR)

发件人： "高文青"<gwqsh@fudan.edu.cn>

收件人： "李继喜"<lijixi@fudan.edu.cn>

抄 送： "李媛媛"<yuanyuan_li@fudan.edu.cn> "潘佳松"<19210700095@fudan.edu.cn> "Suhua Li"<suhua.li@dukekunshan.edu.cn> "qj16@duke.edu"<qj16@duke.edu> "李媛媛"<20110700118@fudan.edu.cn>

Yes

gwqsh

邮箱：gwqsh@fudan.edu.cn

---- Replied Message ----

From	李继喜<lijixi@fudan.edu.cn>
Date	01/06/2024 02:27
To	yuanyuan_li@fudan.edu.cn<yuanyuan_li@fudan.edu.cn>
Cc	潘佳松<19210700095@fudan.edu.cn>,<br>高文青<gwqsh@fudan.edu.cn>,<br>Suhua Li<suhua.li@dukekunshan.edu.cn>,<br>qj16@duke.edu<qj16@duke.edu>,<br>20110700118@fudan.edu.cn<20110700118@fudan.edu.cn>
Subject	Authorships of Cell Death & Disease paper (CDDIS-23-3415RR)

Dear All,

As Dr. Lu Geng and Prof. Jin Ding contributed to the revised paper (CDDIS-23-3415RR), I would like to add them as co-authors.
If you agree, please reply the email with "YES".
Thank you.

李继喜 lijixi@fudan.edu.cn 实名用户 2024/01/06

Re: Authorships of Cell Death & Disease paper (CDDIS-23-3415RR)

发件人： "潘佳松"<19210700095@fudan.edu.cn>

收件人： "李继喜"<lijixi@fudan.edu.cn>

Yes

---- Replied Message ----

From	李继喜<lijixi@fudan.edu.cn>
Date	01/05/2024 23:27
To	yuanyuan_li@fudan.edu.cn<yuanyuan_li@fudan.edu.cn>
Cc	潘佳松<19210700095@fudan.edu.cn>,<br>高文青<gwqsh@fudan.edu.cn>,<br>Suhua Li<suhua.li@dukekunshan.edu.cn>,<br>qj16@duke.edu<qj16@duke.edu>,<br>20110700118@fudan.edu.cn<20110700118@fudan.edu.cn>
Subject	Authorships of Cell Death & Disease paper (CDDIS-23-3415RR)

Dear All,

As Dr. Lu Geng and Prof. Jin Ding contributed to the revised paper (CDDIS-23-3415RR), I would like to add them as co-authors.
If you agree, please reply the email with "YES".
Thank you.

Jixi

Jixi Li, PhD & Professor
School of Life Sciences
Fudan University

Re: Authorships of Cell Death & Disease paper (CDDIS-23-3415RR)

发件人： "李媛媛"<yuanyuan_li@fudan.edu.cn>

收件人： "李继喜"<lijixi@fudan.edu.cn>

Yes

> On Jan 5, 2024, at 23:27, 李继喜 <lijixi@fudan.edu.cn> wrote:
>
> Dear All,
>
> As Dr. Lu Geng and Prof. Jin Ding contributed to the revised paper (CDDIS-23-3415RR), I would like to add them as co-authors.
> If you agree, please reply the email with "YES".
> Thank you.
>
> Jixi
>
>
> ------------------------------
> --

> Jixi Li, PhD & Professor
> School of Life Sciences
> Fudan University
> Room B201, The Biological Bldg, 2005 Songhu Rd.
> Shanghai, China 200438
> Tel:+86-21-31246538 (L); 31246539 (O)
> Email: lijixi@fudan.edu.cn

>

李继喜 lijixi@fudan.edu.cn 实名用户 2024/01/06

Yes!

获取 Outlook for iOS

...

QJ 你自己
qizhou.jiang@dukekunshan.edu.cn

收件人: 李继喜 lijixi@fudan.edu.cn
yuanyuan_li@fudan.edu.cn

抄送: 潘佳松 19210700095@fudan.edu.cn
高文青 gwqsh@fudan.edu.cn
Suhua Li suhua.li@dukekunshan.edu.cn
20110700118@fudan.edu.cn

1月6日 星期六, 10:23

Yes.

获取 Outlook for iOS

...

全部答复

邮件 日历 源 应用

9:15

[illegible]
suhua.li@dukekunshan.edu.cn

收件人: 李继喜 lijixi@fudan.edu.cn

1月6日 星期六, 下午 9:14

Yes

获取 Outlook for iOS

发件人: 李继喜 <lijixi@fudan.edu.cn>
发送时间: 星期五, 一月 5, 2024 11:27 下午
收件人: yuanyuan_li@fudan.edu.cn <yuanyuan_li@fudan.edu.cn>
抄送: 潘佳松 <19210700095@fudan.edu.cn>; 高文青 <gwqsh@fudan.edu.cn>; Suhua Li <suhua.li@dukekunshan.edu.cn>; Qizhou Jiang <qizhou.jiang@dukekunshan.edu.cn>; 20110700118@fudan.edu.cn <20110700118@fudan.edu.cn>
主题: Authorships of Cell Death & Disease paper (CDDIS-23-3415RR)

Dear All,

As Dr. Lu Geng and Prof. Jin Ding contributed to the revised paper (CDDIS-23-3415RR), I would like to [illegible]

全部答复

邮件 搜索 6 日历
